# β-Cell Death in Diabetes: Past Discoveries, Present Understanding, and Potential Future Advances

**DOI:** 10.3390/metabo11110796

**Published:** 2021-11-22

**Authors:** Noyonika Mukherjee, Li Lin, Christopher J. Contreras, Andrew T. Templin

**Affiliations:** 1 Department of Biochemistry and Molecular Biology, Indiana University School of Medicine, Indianapolis, IN 46202, USA; nmukher@iu.edu; 2 Lilly Diabetes Center of Excellence, Indiana Biosciences Research Institute, Indianapolis, IN 46202, USA; llin@indianabiosciences.org (L.L.); chcontre@iu.edu (C.J.C.); 3 Department of Medicine, Roudebush Veterans Affairs Medical Center, Indiana University School of Medicine, Indianapolis, IN 46202, USA; 4 Center for Diabetes and Metabolic Diseases, School of Medicine, Indiana University, Indianapolis, IN 46202, USA

**Keywords:** β cell, cell death, islet, diabetes mellitus, cytotoxicity

## Abstract

β-cell death is regarded as a major event driving loss of insulin secretion and hyperglycemia in both type 1 and type 2 diabetes mellitus. In this review, we explore past, present, and potential future advances in our understanding of the mechanisms that promote β-cell death in diabetes, with a focus on the primary literature. We first review discoveries of insulin insufficiency, β-cell loss, and β-cell death in human diabetes. We discuss findings in humans and mouse models of diabetes related to autoimmune-associated β-cell loss and the roles of autoreactive T cells, B cells, and the β cell itself in this process. We review discoveries of the molecular mechanisms that underlie β-cell death-inducing stimuli, including proinflammatory cytokines, islet amyloid formation, ER stress, oxidative stress, glucotoxicity, and lipotoxicity. Finally, we explore recent perspectives on β-cell death in diabetes, including: (1) the role of the β cell in its own demise, (2) methods and terminology for identifying diverse mechanisms of β-cell death, and (3) whether non-canonical forms of β-cell death, such as regulated necrosis, contribute to islet inflammation and β-cell loss in diabetes. We believe new perspectives on the mechanisms of β-cell death in diabetes will provide a better understanding of this pathological process and may lead to new therapeutic strategies to protect β cells in the setting of diabetes.

## 1. Introduction

Diabetes mellitus is a global epidemic that afflicts over 450 million people worldwide, and this number is expected to rise to nearly 700 million by 2045 [1]. It costs an estimated USD 850 billion annually [1] and is a risk factor for other morbidities, including cardiovascular disease, chronic kidney disease, stroke, blindness, and amputation [2]. Diabetes is defined clinically by the presence of hyperglycemia [3], which is largely caused by insufficient insulin release from pancreatic β cells [4]. It can be broadly classified into type 1 diabetes, which involves the autoimmune-associated destruction of β cells, and type 2 diabetes, which is characterized by insulin resistance and a relative insufficiency of β-cell function and mass [5,6]. In addition to these major classifications of diabetes, gestational diabetes, monogenic diabetes, latent autoimmune diabetes in adults, diseases of the exocrine pancreas, and drug- and chemically-induced diabetes have also been identified in individuals who present with hyperglycemic phenotypes distinct from type 1 or type 2 diabetes [7].

β-cell death is a key event in the pathogenesis of diabetes. To summarize and integrate our current understanding of β-cell death in both major forms of this disease, we have conducted a literature review that includes information from books, book chapters, clinical standards of care guidelines, diabetes incidence databases, and journal articles spanning from the late 1800s to the present day. In this review, we aim to (1) summarize previously published data on the presence of and mechanisms underlying β-cell death in diabetes, (2) discuss how these findings have shaped our current understanding of the role of β-cell death in diabetes pathogenesis, and (3) identify knowledge gaps and areas of research that may lead to new strategies to protect β cells in the setting of diabetes. We begin by summarizing the initial discoveries that led to our understanding of β-cell loss in insulin insufficiency and hyperglycemia in diabetes, with a specific focus on studies conducted in humans or human tissues.

## 2. Understanding β-Cell Death in the Pathogenesis of Diabetes Mellitus

### 2.1. Early Observations of Insulin Deficiency as a Driver of Hyperglycemia in Diabetes

The presence of excess glucose in the urine of individuals with diabetes has been recognized for centuries [8], but it was not until the pioneering experiments of von Mering and Minkowski around 1890 that the pancreas was widely recognized to regulate blood glucose concentrations [9]. Prior to this observation, Paul Langerhans observed in the 1860′s that “islands” of cells were present in the pancreas, and that these cells were distinct from the surrounding exocrine tissue [10]. In 1901, Eugene Opie observed that diabetes mellitus was related to the presence of “hyaline degeneration” of the islands of Langerhans [11], and in 1907, M. A. Lane showed that the pancreas “islet” areas described by Langerhans were composed of at least two unique cell types [12]. These observations suggested that a molecule synthesized by islets was responsible for maintaining glucose homeostasis, and that deficiency of this molecule (which we now recognize as insulin) was associated with diabetes. In 2021, we celebrated the 100-year anniversary of Banting and Best’s discovery that an “internal secretion” of the pancreas could be isolated and administered to diabetic subjects to reduce hyperglycemia [13,14]. In 1922, Leonard Thompson became the first person to receive insulin therapy for diabetes [15], and shortly thereafter commercial production and wide-spread use of life-saving insulin began [16]. Together, these early works identified loss of insulin as a key driver of the hyperglycemia in diabetes mellitus and led to a new stage of research focused on understanding the pathogenic factors that lead to insulin-deficiency and diabetes.

### 2.2. Observations of β-Cell Loss in Human Diabetes

Although loss of insulin production was understood to be a factor in the development of diabetes by the early 1920s, it was at first not clearly established that a loss of functional β cells led to insufficient insulin release. Arriving at this conclusion required a greater understanding of the divergent diabetic phenotypes observed clinically [17,18]. It was traditionally accepted that diabetes generally occurs in two distinct populations: (1) children and young adults with normal or low body weight, and (2) aging and overweight adults. It was also recognized that diabetic phenotypes in these groups were similarly dichotomous, with hyperglycemia normally appearing as an acute and fatal affliction in children, but as a manageable chronic condition in adults [19]. We now consider these divergent phenotypes to be separate diseases: type 1 diabetes (T1D, also known as insulin-dependent diabetes and formerly as juvenile diabetes), which is associated with islet autoimmunity and near complete loss of insulin production [17,20,21]; and type 2 diabetes (T2D, also known as insulin-independent diabetes), which is associated with insulin resistance and relative insulin insufficiency [18,22,23]. In this section, we review important research findings that underlie our current understanding of β-cell loss in both major forms of diabetes mellitus in humans.

#### 2.2.1. β-Cell Loss in T1D

To better understand the factors that contribute to loss of insulin production in diabetes, pathologists began to study pancreas sections from diabetic and non-diabetic individuals collected at autopsy well over one hundred years ago. Following the early works by Opie, Lane, and others discussed above, additional studies began to describe diabetes-associated β-cell loss in greater detail. Among these are the seminal studies conducted by Maclean and Ogilvie in the 1950s. The histological examination of pancreas sections, along with measurement of tissue mass, from young and old individuals (but primarily subjects over 40 years of age), revealed a significant reduction in beta cell mass in individuals with diabetes [18]. Among diabetic subjects, β-cell mass was observed to be lower in individuals diagnosed at less than 25 years of age and higher in individuals diagnosed later in life [18]. When Maclean and Ogilvie analyzed pancreas tissue sections specifically from young subjects, they found diabetic individuals exhibit reduced islet number and mass, but no difference in islet size compared to non-diabetic individuals [24]. They also observed that among young diabetic subjects, those with long-standing (chronic) disease have less islet mass than those with short-standing (acute) disease [24]. When pancreas sections from young subjects with “juvenile diabetes” were analyzed by Doniach and Morgan in 1973, they were found to have an 85% reduction in the number of islets present [25]. Stefan and colleagues compared pancreas sections from 13 non-diabetic subjects and 2 insulin-dependent diabetic subjects (T1D), and observed that the primary difference between these groups was the reduced number of insulin-producing β cells [26]. When Klöppel et al. compared pancreas samples from recently diagnosed T1D individuals with age-matched controls in 1984, a 70–85% reduction in β-cell abundance was again revealed [27]. These observations of reduced β-cell and islet abundance were among the first to demonstrate β-cell loss in what we now refer to as T1D.

#### 2.2.2. β-Cell Loss in T2D

In addition to observations of β-cell loss in T1D, evidence for β-cell loss in T2D has also existed for decades. In 1901, Eugene Opie observed the relationship between diabetes mellitus, islet cell loss, and the presence of islet “hyaline deposits”, the last of which are now recognized as islet amyloid deposits [11,28]. Notably, Maclean and Ogilvie excluded from their 1955 analyses pancreas samples that exhibited hyalinization of islets, which were observed in tissue specimens of individuals of 40 years of age and older [18]. In contrast to their observations of extensive β-cell loss in T1D, Maclean and Ogilvie observed a less severe reduction in β-cell mass in individuals with T2D [18]. A more recent study estimates that a ~35% reduction in β-cell mass is present in T2D [29].

It is important to note that controversy has surrounded the question of whether loss of β-cell mass truly contributes to the pathogenesis of T2D. Indeed, one early histological observation found increased islet size in a hyperglycemic individual [30]. Separately, Ogilvie found that islet size was increased in 19 obese individuals versus controls, although none of these subjects had diabetes [31]. These observations likely reflect the presence of insulin resistance in obesity, leading to a compensatory increase in islet size [32]. In 1985, Klöppel et al. noted that while T1D results from selective loss of β cells, these insulin-producing cells are always present in T2D [5], and Guiot et al. also questioned whether reduced β-cell mass was responsible for insulin insufficiency in T2D [33]. A more recent study compared β-cell volume in explanted human pancreas tissue slices and found no significant reduction in β-cell volume in T2D versus control donors [34].

On the other hand, several studies have observed significant reductions in β-cell abundance in adult diabetics compared to non-diabetic subjects. One differential volumetric analysis of pancreatic islets revealed a β-cell deficit in adults with diabetes that was associated with increased maximal blood glucose, suggesting that β-cell loss leads to reduced insulin production in T2D [35]. The examination of human pancreas from normoglycemic and T2D subjects following autopsy revealed a decrease in β cells in individuals with hyperglycemia [18]. In a cross-sectional analysis of pancreas sections, Sakuraba et al. observed a 30% reduction in β-cell mass in T2D subjects from Japan, along with increased amyloid deposition and oxidative stress compared to normoglycemic controls [36]. Butler and colleagues also sought to determine whether decreased β-cell mass underlies the hyperglycemia observed in T2D, when in 2003 they analyzed 124 pancreas tissue sections collected at autopsy. They found that individuals with impaired fasting glucose (IFG) or T2D exhibited 40% and 63% reductions, respectively, in β-cell volume compared to non-diabetic individuals [37]. Jurgens et al. analyzed 68 pancreas tissue samples and again observed a decreased β-cell area in tissue from individuals with T2D [38].

Given the available data from individuals with T2D, it is now widely believed that a relative deficit of β-cells exists in this disease. The recently appreciated heterogeneity of T2D phenotypes and human β-cell mass [39,40,41,42] may help explain the ongoing debate as to the role of β-cell loss in T2D pathogenesis.

### 2.3. Loss of β-Cell Function in Human Diabetes

As well as evidence for β-cell loss derived from histological measurements of pancreas tissue samples, reduced β-cell function is present in both major forms of diabetes. In general, these studies have defined impaired β-cell function as (1) impaired insulin synthesis leading to reduced insulin content in islets, (2) reduced plasma insulin (or C-peptide) in the fed or fasted state, or (3) reduced acute insulin release in response to a glucose bolus or mixed meal challenge [4,43]. Using such measurements, numerous studies have identified loss of β-cell function as a primary factor underlying the hyperglycemia that characterizes T1D and T2D.

#### 2.3.1. β-Cell Dysfunction in T1D

Given the use of insulin as a treatment for T1D, the loss of insulin production in diabetes was widely recognized in the 1920s. In 1952, Wrenshall and colleagues showed that less extractable insulin was present in pancreases from individuals with diabetes compared to those without diabetes, and that, among those with diabetes, individuals diagnosed at less than 20 years of age possessed significantly less insulin than individuals diagnosed at 20 years of age or older [17]. Later, Cerasi and Luft used IV glucose infusion tests and computer modeling to identify an insufficient insulin response to glucose as a characteristic of diabetic individuals [44], concluding that this loss of insulin secretion was likely a “prerequisite” for diabetes. More recently, a large clinical study analyzed fasting C-peptide data collected from 3668 subjects at the time of T1D diagnosis [45]. Those diagnosed with T1D at a younger age exhibited lower fasting C-peptide and a greater decline in fasting C-peptide over time [45].

Despite the clear loss of β-cell abundance and function in T1D, however, some functional β cells persist in individuals with this disease. The Medalist Study analyzed 1019 individuals with a duration of T1D over 50 years [46]. Among these individuals, 32.4% had detectable C-peptide levels and 5.8% were able to double C-peptide in response to a mixed meal tolerance test [46], despite their severe insulin insufficiency and hyperglycemia. A potential explanation for this residual insulin secretory function was uncovered when β-cell lineage tracing experiments identified dedifferentiation as an important driver of β-cell loss in β-cell specific FoxO1 knockout mice [47]. Later, a population of β cells that survive autoimmune attack was identified in mouse and human islets, with these cells expressing reduced β-cell identity genes and arising in response to insulitis and inflammation [48]. These studies suggest that a subpopulation of β cells exist that, while not fully functional, are protected from cell death in the context of T1D.

#### 2.3.2. β-Cell Dysfunction in T2D

Insufficient insulin secretion in T2D, and its relationship to obesity, insulin resistance, and aging, are also well documented. In 1963, Karam and colleagues observed increased insulin responses in obese versus normal weight subjects following a glucose injection [49]. In 1967, Perley and Kipnis observed that, in response to either oral or IV glucose, plasma insulin was higher in obese versus normal weight individuals without diabetes [22]. Later, Bonadonna and colleagues again found that insulin secretion was increased in obese versus control subjects and noted that obese individuals displayed decreased glucose uptake in response to specified insulin concentrations, that is, they were insulin resistant [23]. Such observations established insulin resistance and hyperinsulinemia as characteristics of obese, non-diabetic individuals.

In contrast, Perley and Kipnis observed that obese diabetic subjects failed to increase insulin secretion to levels similar to their obese non-diabetic counterparts, displaying a “marked impairment in insulin secretion” [22]. More recently, Mitrakou et al. found that glucose intolerant individuals displayed reduced insulin secretion following glucose ingestion compared to age and weight matched controls subjects [50]. Of note, the reduced insulin secretion observed in T2D is not confined to obese individuals. Similar observations of insulin resistance and β-cell dysfunction have been made in aged individuals with normal body weight and glucose intolerance [51]. More recent observations have clearly defined the relationship between insulin resistance, β-cell function, and T2D [52]. These studies demonstrate that obese, non-diabetic individuals increase insulin release to maintain normal blood glucose in the face of insulin resistance, and that this compensatory insulin response is lost in hyperglycemic diabetic individuals.

Together, these data provide evidence that loss of β-cell abundance and function are primary elements contributing to the insulin insufficiency and hyperglycemia that characterize both major forms of diabetes. Understanding the specific factors that underlie β-cell loss in these diseases may lead to new approaches to maintain insulin secretion and thereby treat or cure diabetes.

### 2.4. Observations of β-Cell Death in Human Diabetes

β-cell abundance is regulated by various processes, including proliferation (division of existing β cells), neogenesis (generation of new β cells from progenitor cells), dedifferentiation (transformation of β cells to other cell types), and β-cell death (destruction of β cells, often referred to as apoptosis). Although each of these mechanisms contribute to the prevailing β-cell mass present in an individual, increased β-cell death is regarded as a primary factor underlying β-cell loss in diabetes [53,54]. In this section, we review evidence for β-cell death as a driver of reduced β-cell mass in human T1D and T2D.

#### 2.4.1. Evidence for β-Cell Death in Human T1D

Evidence for β-cell death in human diabetes is derived in large part from histological comparisons of pancreas sections from diabetic and non-diabetic subjects collected at autopsy. Initial observations of β-cell death in diabetes focused on the pathogenesis of T1D and relied on indirect evidence; although numerous studies were able to convincingly demonstrate β-cell loss in the context of T1D (as discussed previously), and β-cell death was believed to underlie these observations, direct evidence for increased rates of β-cell death was lacking [53]. Following the advent of histochemical methods to identify dead and dying cells in the 1990s [55,56,57], direct evidence for β-cell death in diabetes emerged. In 2005, Meier and colleagues compared pancreas section from 42 individuals with T1D with those from 14 non-diabetic individuals [58]. They observed that the rate of β-cell death was doubled in T1D versus control subjects, and that this was accompanied by an increased presence of islet macrophages and T lymphocytes [58]. In 2007, Buter et al. examined pancreas samples collected from individuals with recent-onset T1D and age-matched control subjects and found that β-cell death was increased in T1D while β-cell proliferation was unchanged [37]. These data support immune-associated β-cell death as a contributor to β-cell loss in T1D.

#### 2.4.2. Evidence for β-Cell Death in Human T2D

In T2D, β-cell death has similarly been postulated to underlie the relative loss of β-cells, although little direct evidence in diabetic human subjects was available before the start of the 21st century [54,59]. Subsequently, histological examinations of pancreas samples have shown an association between increased β-cell death and the relative loss of β-cell mass and function that characterize human T2D. In their 2003 study of 124 pancreas samples, Butler and colleagues observed increased β-cell death in subjects with T2D, with rates being increased 10-fold in lean individuals with T2D and 3-fold in obese individuals with T2D compared to weight-matched non-diabetic controls [37]. Notably, no alterations in β-cell proliferation or neogenesis were observed between weight-matched diabetic and non-diabetic subjects, but they observed increased islet amyloid deposition in both lean and obese subjects with T2D [37]. When Jurgens and colleagues examined pancreas sections from 29 diabetic and 39 non-diabetic subjects in 2011, they also observed increased rates of β-cell death in individuals with T2D [38]. In addition, they found that islet amyloid deposition was higher in those with T2D and that the degree of islet amyloid formation was positively associated with β-cell death and negatively associated with islet insulin area [38]. In sum, these findings indicate that β-cell mass is decreased in T2D at least in part, due to increased β-cell death.

#### 2.4.3. Considerations for Quantifying β-Cell Death in Human Diabetes

If increased β-cell death accounts for the decreased β-cell mass in diabetes, why is it that β-cell death is a relatively rare histological event? Numerous studies have reported “low” rates of β-cell death in diabetic subjects, with roughly one dead β cell present in every 2–3 diabetic islets [37,38,60]. It is noted that these measurements represent only a snapshot in time, and that in situ dead islet cells are phagocytosed by islet-resident macrophages. Therefore, the true frequency of β-cell death in diabetes, and how accumulation of this death translates to β-cell loss over time, is difficult to determine in tissue samples.

In the last decade, new methodologies have enabled detection of β-cell death through the quantification of unmethylated insulin DNA from blood samples. In short, unmethylated insulin gene promoter DNA was shown to be present specifically in β cells, but not other tissue types [61,62]. Various DNA amplification methods have been used to amplify and quantify the presence of such DNA, with increases in the quantity this β-cell derived DNA serving as a proxy measure of in vivo β-cell death [61,62,63]. In 2011, Akirav et al. reported a method for detecting unmethylated insulin (INS) DNA in serum and observed that presence of unmethylated INS DNA was specific to islet β cells [61]. In addition, they found increased quantities of unmethylated INS DNA in serum collected from subjects with new-onset T1D [61]. Fisher and colleagues determined that both unmethylated and methylated INS DNA were increased in individuals with new-onset T1D, and that unmethylated (but not methylated) INS DNA remained elevated at 8 weeks-post-onset [62]. They also found that unmethylated INS DNA was elevated in adult compared to pediatric non-diabetic subjects, but that individuals with T2D did not exhibit elevated serum unmethylated INS DNA compared to aged-matched non-diabetic controls [62]. Husseiny et al. found that unmethylated INS DNA was also elevated following islet transplant [63]. Other recent studies have largely confirmed these initial observations and indicate that increased rates of β-cell death occur in T1D, aging, and islet transplantation [64,65].

## 3. Advances in Understanding the Mechanisms of β-Cell Death in Diabetes

Although studies in human subjects are of utmost importance in our search to identify the mechanisms that cause β-cell loss in diabetes, the nature of human studies limits what types of experiments can be performed. As such, researchers must also apply in vitro and in vivo studies of animal models of diabetes. These studies provide important insights into the pathogenesis of diabetes that can be further interrogated in humans and may lead to new therapeutic approaches in human disease.

Similar to observations made in diabetic human subjects, animal models of diabetes exhibit increased rates of β-cell death. In 1997, O’Brien et al. described an increased presence of dead β cells in the non-obese diabetic (NOD) mouse model of autoimmune diabetes [66]. Around the same time, Augstein et al. demonstrated that islet cell death was associated with β-cell loss and deterioration of glycemic control in NOD mice [67]. Increased rates of β-cell death were also observed in rats that spontaneously develop diabetes, but not in diabetes resistant rats [68]. β-cell death has also been reported to underlie loss of insulin secretory capacity and hyperglycemia in mouse models of T2D. Donath et al. observed increased rates of β-cell death in association with increased blood glucose in a sand rat model of T2D [69]. Similarly, increased β-cell death contributes to the reduced β-cell mass in Zucker diabetic fatty rats [70,71].

**Figure 1:**
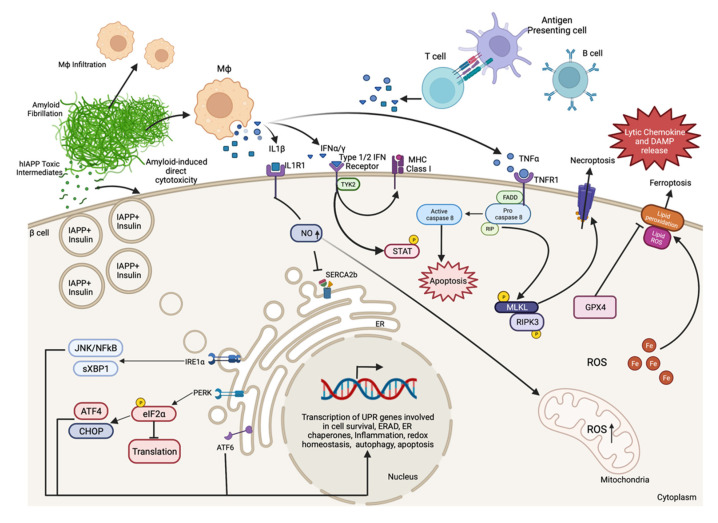
Schematic model of diabetes-relevant mechanisms of β-cell death. Several mechanisms of β-cell death discussed in this review, including autoimmune-associated, islet amyloid-induced, proinflammatory cytokine-mediated, and ER stress- and oxidative stress-induced β-cell death are illustrated in this diagram. This model represents an integrated, but simplified and incomplete graphical representation of diabetes-associated mechanisms of β-cell death.

Indeed, numerous animal models of diabetes display β-cell death and can be used to examine cell death signaling pathways in greater detail. Several factors may contribute to β-cell death during the progression of diabetes, some of which include β-cell autoimmunity, increased islet amyloid deposition, proinflammatory cytokine signaling, endoplasmic reticulum (ER) stress, oxidative stress, and elevated fatty acid and/or glucose concentrations (Figure 1). Many of these mechanisms of β-cell death are related, and each has been reviewed in detail elsewhere [6,59,72,73,74,75,76,77,78]. In the following section, we review some important research findings from humans and animal models that have advanced our understanding of the molecular mechanisms that promote β-cell death in diabetes.

### 3.1. Autoimmune-Associated β-Cell Death in T1D

Upon examination of pancreas sections from 42 individuals with T1D and 14 non-diabetic controls, Meier and colleagues observed that β-cell death was twice as frequent in individuals with T1D [58]. They also observed that this increased cell death was accompanied by increased infiltration of macrophages and T lymphocytes in islets, suggestive of autoimmune-mediated destruction of β cells in T1D [58]. Coppieters et al. identified islet-autoreactive CD8+ T cells in insulitic lesions that were specific to T1D patients and were not observed in cases of T2D or gestational diabetes [79]. Research conducted at the level of individual islets suggests β-cell killing is mediated by direct CD8+ T cell contact with β cells, and that M1 polarization of islet resident macrophages by CD4+ T cells contributes to this process [79,80]. Such findings have added to previous observations of autoimmune-associated destruction of β cells in T1D [21,53,81] and led to studies of the mechanisms that promote this process.

#### 3.1.1. The Immune System in T1D-Associated β-Cell Death

T1D is traditionally thought to result from the destruction of healthy β cells by self-reactive T cells that occurs by mistake, a view adopted following identification of islet-reactive CD4+ and CD8+ T cells in individuals with T1D [79,82,83,84]. In line with this understanding, numerous immunotherapy trials have been conducted in individuals with recent-onset T1D in the hope of preserving β-cell mass and function. Teplizumab and otelixizumab, anti-CD3 monoclonal antibodies that target T cells, have been shown to improve C-peptide responses and reduce exogenous insulin requirements in individuals with recent-onset T1D [85,86,87]. In addition, teplizumab has been shown to delay the onset of T1D in relatives at risk for T1D [88]. Abatacept, a molecule used to modulate T cell co-stimulation, has also been shown to transiently preserve β-cell function in T1D patients [89]. Studies in animal models of diabetes further support the role of T cells in T1D. In 1985, Miyazaki et al. reported on the prominence of CD4+ and CD8+ T cells in the insulitic lesion observed in the NOD mouse model of T1D [90]. In 1993, Christianson and colleagues observed that while NOD background mice lacking T and B cells (NOD/SCID) did not develop diabetes, diabetes could be induced in these mice by adoptively transferring T cells from diabetic NOD mice [91]. Around this time, Posselt and associates also observed that autoimmune diabetes in spontaneously diabetic BioBreeding rats could be prevented by intrathymic islet transplantation, possibly due to modulation of the T cell repertoire upon exposure to transplanted islet antigens [92]. In sum, these studies provide strong evidence for T cell-associated β-cell death in T1D.

In 1974, autoantibodies against islet cell proteins were first detected in the sera of T1D patients [81]. Autoantibodies observed in individuals with T1D include those against islet cell cytoplasmic protein (ICA), insulin (IAAs), glutamic acid decarboxylase (GAD65), insulinoma antigen 2 protein (IA-2), and zinc transporter 8 (ZnT8) [93]. Presence of at least one of these autoantibodies is present in >95% of individuals diagnosed with T1D [93]. The most well-known β-cell-specific antigens are insulin and pro-insulin, with insulin autoantibodies (IAAs) detected in >59% patients with late pre-clinical or early-onset T1D [94]. Despite the strong association between islet autoantibodies and T1D, it is debated whether autoantibodies drive β-cell death or whether they arise because of islet destruction. However, it is largely accepted that the presence of islet autoantibodies is associated with an increased risk of developing T1D [95]. Rituximab, an anti-CD20 monoclonal antibody that depletes B lymphocytes was observed to delay, but not prevent, loss of β-cell function in subjects with recently diagnosed T1D [96]. Mariño et al. observed that B lymphocyte depletion delayed diabetes onset and reduced diabetes incidence in NOD mice, again indicating a role for B cells in T1D pathogenesis [97].

#### 3.1.2. The β-Cell in T1D-Associated β-Cell Death

Given that islet autoimmunity is a key feature of T1D, it is important to identify the factors that initiate this immune-associated β-cell destruction. Counter to the traditional hypothesis that the immune system errantly identifies β cells for removal, another hypothesis has more recently been put forward: that the β cell itself prompts the autoimmune assault observed in T1D. Several observations support the concept that β cells contribute to the pathophysiology of T1D. Studies performed in NOD mice have shown that β-cell death precedes the appearance of insulitis [66] and promotes β-cell autoantigen presentation [98]. NOD mouse islets exhibit increased endoplasmic reticulum (ER) stress markers and alterations in ER structure prior to insulitis and hyperglycemia, suggesting that β-cell stress contributes to development of T1D in this model [99]. Indeed, treatment of prediabetic NOD mice with an ER stress mitigating agent (TUDCA) was shown to reduce diabetes incidence [100]. β-cell stress may lead to the production of antigenic products, such as “hybrid insulin peptides”, which are composed of proinsulin and other secretory peptides and have been identified as targets of CD4+ T cells found in NOD mice and individuals with T1D [101].

Several notable reviews have discussed the role of β cells in their own demise in T1D. In 2009, Eizirik and colleagues discussed an ongoing “dialogue” between immune cells and β cells mediated via cytokine and chemokine signaling, and by immunogenic danger signals released from dying β cells [102]. Several reviews have discussed the association of β-cell stress, dysfunction, and death as predecessors, not only outcomes, of T1D [103,104], with the most recent of these describing β cells as an “active participant” in T1D pathogenesis and not a “non-provoking victim of an autoimmune attack” [105]. Also supporting a role of the β cell in T1D pathogenesis, immunosuppression therapies targeting T and B cells (discussed above) exhibit a lack of durable effects, with these interventions delaying, but not preventing, the development of T1D [85,86,89,96].

These observations suggest that unfounded islet immune responses may not be the inciting event in β-cell death in T1D. Together, they have led the field to question whether β cells, the target tissue of immune attack, may participate in their own demise and carry more blame in T1D pathogenesis than originally thought. Additional studies are needed to better understand how β-cell stress or death may initiate the islet immune responses that have long characterized T1D.

### 3.2. Islet Amyloid-Induced β-Cell Death in T2D

In 1901, Eugene Opie first reported a form of islet “hyaline degeneration” that occurs in association with diabetes mellitus and that we now recognize as islet amyloid deposition [11]. More recent studies have confirmed the relationship between islet amyloid deposition and hyperglycemia in T2D, with these works demonstrating that islet amyloid deposition is associated with reduced β-cell abundance and increased β-cell death in humans [37,38]. Islet amyloid formation is accepted to be a contributor to the pathogenesis of T2D, but studies have suggested that it may also contribute to β-cell loss in T1D [106,107,108] and following islet transplantation [109,110]. Islet amyloid deposits are insoluble fibrillar structures composed primarily of islet amyloid polypeptide (IAPP, also known as amylin), a β-cell secretory product [111,112]. Although monomeric IAPP has physiological functions on satiety and gastric emptying [113,114], human IAPP (hIAPP) is amyloidogenic and can aggregate into larger structures, including oligomers, protofibrils, and eventually full amyloid fibrils [115]. A significant effort has been directed towards identifying the mechanisms that underlie amyloid-associated β-cell death in diabetes.

Early observations of the mechanisms of hIAPP-induced β-cell death focused on the direct actions of hIAPP to induce cytotoxicity [116,117]. Studies of hIAPP have found that oligomeric peptide species can directly elicit β-cell cytotoxicity in cell lines and primary islet cells [118,119,120]. This cytotoxic effect has been linked to both receptor-mediated signaling events [121,122,123,124] and cytotoxic interaction with cell membranes [125,126,127]. For example, the receptor for advanced glycation end-products (RAGE) has been shown to mediate hIAPP-induced β-cell death, with genetic deficiency or pharmacological inhibition of RAGE reducing hIAPP-induced β-cell loss and β-cell death [123]. Alternatively, in 1994 Lorenzo et al. showed that hIAPP is toxic to primary rat and human β cells, proposing that this toxicity was mediated by a direct contact of hIAPP with the cell surface [116]. Later, oligomeric hIAPP species were found to disrupt cell membranes via pore formation [128]. In addition to the direct action of hIAPP on β cells, it has been demonstrated that hIAPP also induces cytokine production from macrophages and dendritic cells via the activation of the NLRP3 inflammasome [129,130]. Work to define the structure of these cytotoxic hIAPP species is ongoing [128,131,132]. A better understanding of the mechanisms of direct hIAPP-induced cytotoxicity may provide strategies to reduce the β-cell death elicited by hIAPP.

In contrast to hIAPP, rodent IAPP is not amyloidogenic or cytotoxic due to several amino acid substitutions in the amyloidogenic region of the peptide in rodent compared to human IAPP [115]. Thus, rodent models that express hIAPP in the β cell have been created [133,134,135,136]. These animal models develop in situ islet amyloid deposits, β-cell loss, and β-cell death similar to that observed in humans with T2D, thereby facilitating study of the process of islet amyloid-induced β-cell destruction [128,132,133]. Given that IAPP is a β-cell secretory peptide, insulin resistance and high secretory demand were postulated to promote to islet amyloid formation in T2D. Indeed, genetic loss of β-cell secretory function reduces islet amyloid formation [137], while elevated glucose concentrations increase amyloid deposition [138]. More recent studies of these animal models have led to a series of observations that identified amyloid formation as a driver of islet inflammation [139,140,141,142]. In vivo studies using these models showed that islet amyloid deposition is associated with increased cytokine (Il1b, Tnf, Il6), chemokine (Ccl2, Cxcl1), and immune cell marker (Emr1, Itgax) expression [140,141], and several studies identified IL-1β as an important mediator of amyloid-induced β-cell death [130,139,140,142,143]. Studies in other models of islet amyloidosis have identified ER stress as an important contributor to amyloid-induced β-cell death [144,145].

Several strategies to ameliorate hIAPP-induced β-cell death have been evaluated. Various small molecule chemical inhibitors have been shown to effectively block hIAPP aggregation and its associated cytotoxicity [146,147,148], with the same being true of certain endogenous proteins [149,150]. Interestingly, the structure of amyloid aggregates is similar between divergent amyloidogenic peptides, such as hIAPP and amyloid β [151], suggesting that a single amyloid-inhibiting molecule may be used to prevent amyloidosis in multiple tissues, such as the islet and brain [132]. In addition to these approaches, vaccination against hIAPP has been shown to effectively prevent amyloid-associated β-cell loss in some [120,152], but not all [153], studies. Other work has focused on preventing islet amyloid associated inflammation, with blockade of IL-1β and toll-like receptor signaling both shown to be effective in preventing β-cell loss [130,143]. With amyloid-induced cytotoxicity being a well-recognized contributor to β-cell death in T2D, strategies to prevent hIAPP-induced β-cell loss are needed.

### 3.3. Mechanisms of Proinflammatory Cytokine-Induced β-Cell Death

As discussed, proinflammatory cytokines from islet resident or infiltrating immune cells contribute to β-cell loss in both T1D and T2D. A series of investigations have focused on understanding the role of proinflammatory cytokines on β-cell dysfunction and death. These studies have used single cytokines or combinations of cytokines to model the inflammatory environment to which β cells are exposed in the pathogenesis of diabetes. β cells express cytokine receptors that, when activated, initiate complex signaling cascades that can impact insulin secretion [154,155], ER stress [99,156], oxidative stress [157,158,159], and eventually cell death [160,161,162,163]. Treatment with a combination of IL-1β, TNFα, and IFNγ is commonly used to study the mechanisms of cytokine-induced β-cell death. These studies have characterized cytokine-stimulated signaling pathways in β cells and evaluated the impact of these on β-cell death in the pathogenesis of diabetes.

#### 3.3.1. IL-1β Signaling in β-Cell Death

The treatment of primary β cells with interleukin 1β (IL1β) for 24 to 48 h was found to elicit cell death, and these effects could be prevented by the inhibition of nitric oxide synthase, suggesting that IL1β mediates its cytotoxic effect through NO production [157,159,164]. IL1β has also been shown to induce ER Ca^2+^ release and activate ER stress pathways. Cotreatment of immortalized and primary rat β cells with a combination of IL1β and IFNγ has been shown to downregulate SERCA2b, a Ca^2+^ pump important for ER homeostasis, activate ER stress, and induce β-cell death, with these effects again being dependent on NO production [156]. IL1β has also been shown to cause β-cell death via activation of the JNK signaling pathway in rat and mouse β cell lines resulting in cell death [165,166]. Anakinra, a clinically approved IL-1 receptor antagonist (IL-1RA), has been shown to prevent cytokine-mediated NO production, mitochondrial dysfunction, and cell death in cultured rat islets [167]. Overexpression of dominant negative MyD88, an IL-1R interacting protein, was found to decrease nuclear NF-kB localization, NO production, and cell death in an IL-1β treated mouse β-cell line [168].

#### 3.3.2. TNFα Signaling in β-Cell Death

Tumor necrosis factor α (TNFα) has been recognized as a cell death stimulus since the 1970s [169,170]. In 1999, Ishizuka and colleagues showed that TNF receptor 1 (TNFR1) and its signaling components TRADD, FADD, and FLICE are expressed in the MIN6 mouse β-cell line, and that TNFα elicits death in a time- and dose-dependent manner in these cells [171]. To test the role of TNFα in autoimmune diabetes, Green et al. developed an NOD mouse with transgenic expression of TNFα in β cells and observed an accelerated diabetes onset, with β-cell death preceding hyperglycemia and immune cell infiltration in this model [172]. TNFR1 deficient NOD mice developed insulitis similar to control NOD mice, yet they failed to develop diabetes [173]. When TNFR1 deficient mice were subjected to adoptive transfer of spleen cells from diabetic control NOD donor mice, diabetes was significantly delayed, indicating a role for β-cell TNFR1 signaling in autoimmune-mediated β-cell death [173]. TNFα is considered an important β-cell death effector. However, while TNFα alone is capable of eliciting cell death in immortalized β-cell lines, it has been observed that primary β-cell death requires treatment with a combination TNFα and IFNγ [174]. Recently, a human monoclonal antibody targeting TNFα, golimumab, was shown to improve endogenous insulin production and reduce exogenous insulin requirement in individuals with recent onset T1D [175]. This landmark study was the first to directly target TNFα for preservation of β-cell function in human T1D.

#### 3.3.3. IFNγ and IFNα Signaling in β-Cell Death

In 1985, Campbell and associates cultured mouse islets in the presence of interferon γ (IFNγ) and observed a 10-fold increase in major histocompatibility complex (MHC) class I antigen expression on β cells [176]. They also observed that IFNγ directly inhibits β-cell growth and insulin synthesis in RIN-m5F β cells [177]. This marked increase in MHC expression suggested that IFNγ may contribute to β-cell-directed autoimmunity in T1D. However, Thomas et al. found that, while loss of functional IFNγ receptors prevented upregulation of β-cell MHC class I expression, it did not prevent diabetes in NOD mice [178]. Gysemans and colleagues found that loss of signal transducer and activator of transcript-1 (STAT-1), a transcription factor activated by both IFNγ and IFNα, prevents β-cell death in response to IFNγ and IL-1β in vitro, and protected from STZ-induced hyperglycemia in vivo.

Individuals at genetic risk of developing T1D exhibit a type 1 interferon signature in peripheral blood mononuclear cells that precedes the development of islet autoantibodies [179]. IFNα, a type 1 interferon, induces HLA class I expression, ER stress markers, and inflammation in human β cells, and acts in coordination with IL-1β to elicit β-cell death [180]. Polymorphisms in TYK2, a type I interferon receptor signaling molecule, confer risk for the development of diabetes [181]. Mice carrying a mutant Tyk2 fail to respond to type I interferon appropriately, leading to a failed β-cell antiviral response and sensitization to virus-induced diabetes [182]. The loss of TYK2 in human β cells has been shown to reduce IFNα-induced MHC class I upregulation and diminish cell death [183]. Thus, IFNα-TYK2 mediated inflammation and cell death may be required for effective β-cell defense against viral infection.

### 3.4. Endoplasmic Reticulum Stress-Induced β-Cell Death

In the setting of insulin resistance and hyperglycemia, high insulin secretory demand is placed on the β cell. This elevated insulin demand has been described as the β cell’s Achilles’ heel; its innate physiological function of glucose-stimulated insulin secretion makes it vulnerable to chronic ER stress [184]. Although β cells initially activate the unfolded protein response (UPR) to balance the demand for insulin synthesis with the capacity of the ER to process insulin properly [100,185,186], changes in insulin molecule sequence [187], ER Ca^2+^ homeostasis [188,189], or insulin demand [190,191] can favor insulin misfolding that leads to chronic ER stress, and eventually, β-cell death [75,163,166,192]. It has been proposed that ER-stress-mediated protein misfolding may lead to β-cell autoantigen production and adaptive immune responses in T1D [105,193], and contribute to hIAPP aggregation and islet amyloid deposition in T2D [144,145]. The basic molecular mechanisms that govern the UPR and ER stress signaling have been reviewed elsewhere [192,194,195].

#### 3.4.1. ER Stress-Induced β-Cell Death in Models of T2D

Several studies have observed that markers of ER stress are elevated in β cells of humans with T2D and animal models thereof. BiP (binding-immunoglobulin protein, also known as HSPA5) and CHOP (Ddit3, DNA damage inducible transcript 3), two ER stress markers, were found to be increased in β cells from pancreas sections collected from patients with T2D compared to non-diabetic subjects [196]. When Marchetti and colleagues evaluated pancreas sections from subjects with T2D, they observed increased β-cell ER volume and β-cell death compared to non-diabetic subjects [197]. They also observed increased expression of ER stress markers BiP and XBP1 (gene-X-box binding protein 1) when islets from donors with T2D were cultured in high glucose, although this increase was not observed in islets isolated from non-diabetic donors [197]. Huang and associates reported increased CHOP expression in β cells of obese individuals, and increased nuclear localization of CHOP was observed in β cells of obese diabetic compared to obese non-diabetic subjects [144]. In the early 2000s, it was observed that chemical chaperones (which aid protein folding) could reduce ER stress, decrease blood glucose concentrations, and restore insulin sensitivity in an animal model of T2D [190,198]. The importance of β-cell ER stress in this process was highlighted by studies of CHOP, an ER stress-induced transcription factor and key effector of cell death [199,200]. In 2002, CHOP deficiency was found to delay the onset of diabetes in Akita mice that exhibit insulin misfolding and β-cell ER stress [201]. In response to genetic and diet-induced insulin resistance, loss of CHOP resulted in improved blood glucose homeostasis, increased β-cell mass, and decreased β-cell death compared to control mice [202]. The improved metabolic profiles observed in this model of CHOP deficiency were associated with increased expression of islet UPR and oxidative stress response genes [202].

Multiple GWAS studies have been performed to identify gene variants associated with T2D risk, with recent studies finding over 400 genetic loci associated with T2D [203,204]. Among the genes identified in these studies are endoplasmic reticulum to nucleus signaling 1 (ERN1, also known as IRE1α) and wolframin ER transmembrane glycoprotein (WFS1), both of which are involved in ER homeostasis and have been linked to caspase activation and β-cell death [205,206]. The loss of the UPR sensor IRE1α in NOD mice triggers dedifferentiation of β cells and protects them from immune assault [207], and the pharmacological inhibition of IRE1α activation reduces β-cell death and reverses T1D in NOD mice [208].

#### 3.4.2. ER Stress-Induced β-Cell Death in Models of T1D

Although ER-stress-induced β-cell death was initially thought to pertain mostly to T2D, ER stress has since become considered a likely contributor to β-cell destruction in T1D as well. As noted previously, cytokines were found to alter ER Ca^2+^ homeostasis and induce ER stress in vitro [156]. This observation led to in vivo studies on the role of ER stress in T1D. In 2012, Tersey and colleagues observed increased expression of ER stress markers in islets of prediabetic NOD mice compared to non-diabetic control mice, noting that this stress signature preceded insulitis [99]. Using a mouse β-cell line and primary mouse islets, they also observed that proinflammatory cytokine treatment altered ribosomal occupancy of RNA, consistent with translational repression and ER stress [99]. Around the same time, Engin et al. observed that ATF6 and XBP1 expression was deficient in islets from humans with T1D and mouse models of autoimmune diabetes [100]. They also showed that treatment of prediabetic mice with a chemical inhibitor of ER stress increased expression of UPR markers, decreased β-cell death, and reduced T1D incidence [100]. More recently, proinflammatory cytokines have been shown to induce ER stress and cell death in induced pluripotent stem cell-derived β-like cells [163].

Additional evidence for the involvement of ER stress in insulin-dependent diabetes comes from individuals with Wolcott–Rallison syndrome. Here, mutations in eukaryotic translation initiation factor 2-alpha kinase 3 (also known as PERK), which plays a major role in remediating ER stress, are linked to insulin-dependent diabetes that occurs neonatally or in early infancy [209]. Other genetic evidence also points to a contribution of the ER in β-cell death. ER stress contributes to monogenic insulin-dependent diabetes in the case of Wolfram Syndrome, caused by pathogenic variants of the WFS1 gene [210]. WFS1 is localized in the ER membrane, regulates the UPR through its interaction with ATF6 [75], and controls ER and cellular calcium homeostasis [211]. Through impairment of these actions, WFS1 mutations leads to β-cell death [205]. These studies and others indicate a clear importance of maintaining ER homeostasis in β-cell health and the ability for chronic ER stress to induce β-cell death.

### 3.5. Oxidative-Stress-Induced β-Cell Death

Several factors contribute to exposure of β cells to reactive oxygen species (ROS) and oxidative stress. Glucose-stimulated insulin secretion by the β cell requires oxidative phosphorylation to produce ATP and, because reactive oxygen species (ROS) generation is a by-product of mitochondrial respiration, this process is an important source of ROS in β cells [212]. Excess insulin demand can therefore lead to elevated ROS production downstream of increased ATP synthesis [76]. In addition, IL-1β produced from islet resident immune cells can upregulate inducible nitric oxide synthase (iNOS) leading to β cell NO production [157,164]. IL-1β alone or in combination with TNFα has been shown to elicit NO production from primary islet cells and β cell lines, resulting in DNA cleavage, nuclear shrinkage, chromatin condensation, and formation of apoptotic bodies, indicative of apoptosis [213]. Thioredoxin-interacting protein (TXNIP) is a redox regulatory protein that is upregulated in β cells in response to glucose and IFNγ [214,215,216], and has been shown to promote β-cell death [214,217,218].

Despite the propensity of β cells for oxidative stress, enzymes involved in antioxidant defense are expressed at low levels in β cells [219,220]. Primary rat islets and β-cell lines are 20 times more sensitive to peroxide radicals than liver or kidney cells [221], and 10 times more sensitive to hydrogen peroxide than macrophage cell lines [222]. Grankvist et al. determined that the sensitivity of islets to alloxan is due to deficiency of endogenous enzymes protecting against superoxide radicals [223]. It was found that islet antioxidant enzymes, superoxide dismutase (SOD), glutathione peroxidase and catalase were expressed at levels ~30% of that measured in liver [224,225]. Such evidence led to the conclusion that β cells are particularly sensitive to damage induced by ROS. In this sense, β cells might again be viewed as playing a role in their own demise.

An important strategy to protect β cells from oxidative damage is inhibition of the free radical formation that results from a hyperglycemic or hyperlipidemic environment. Tersey and colleagues found that loss of 12-lipoxygenase (12-LO), an enzyme involved in the oxidation of arachidonic acid to proinflammatory intermediates, increases the expression of islet antioxidant enzymes Sod1 and Gpx1, and protects against hyperglycemia in response to a high fat diet [226]. Chemical inhibition of 12-LO reduced ROS production in mouse and human islets in vitro, and inhibition of 12-LO in vivo reduced islet oxidative stress, improved blood glucose, and preserved β-cell mass in STZ-treated mice [227]. Given these finding, approaches to reduce β-cell oxidative stress appear to be promising interventions to maintain functional β-cell mass in the context of diabetes.

### 3.6. Glucotoxicity and Lipotoxicity in β-Cell Death

Understanding the toxic effects of glucose and lipids on β cells have been areas of research interest for several decades. The terms glucotoxicity and lipotoxicity were coined to refer to these phenomena, with the term glucolipotoxicity describing the coordinate action of these two entities to elicit β-cell dysfunction and death. In 1996, Prentki and Corkey proposed that molecules that regulate fatty acid synthesis may act as integrated fuel sensors in various diabetes relevant tissues, including the β cell, muscle, liver, and adipose [228]. They further proposed that, when excess glucose and lipid were both present, “metabolic abnormality, which may be termed glucolipoxia, become(s) apparent” [228]. In this section, we review evidence for glucotoxicity and lipotoxicity as separate entities.

In 1992, Robertson and associates reported that chronic long-term growth of hamster β cells in high glucose media led to reduced insulin mRNA expression and protein content, along with reduced insulin release in response to glucose challenge [229]. Shortly after, it was determined that decreased insulin gene transcription contributes to the diminished insulin synthesis observed in response to chronic high glucose [230]. Later, Gleason et al. observed that the observed reductions in insulin gene expression, protein content, and secretion following growth in high glucose could be reversed after a culture in low glucose media [231]. In 2005, Harmon and associates showed that glucose-associated oxidative-stress causes reductions in Pdx1 and MafA expression, which in turn contribute to diminished insulin gene transcription [232]. More recently, reduction of β-cell insulin secretory demand (or β-cell “rest”) has been shown to restore mature insulin content and biphasic glucose-stimulated insulin secretion [233].

Chronically elevated levels of free fatty acids (FFAs) have also been shown to exert toxic effects on β cells, a phenomenon termed lipotoxicity [77]. In the early 1990s, it was observed that prolonged exposure of rat islets to FFAs leads to reduced insulin synthesis and GSIS, but increased basal insulin release in vitro [234,235]. Prolonged treatment of human islets with FFAs reduced GSIS [236] and was shown to induce β-cell apoptosis, with cell death being caspase and ceramide dependent [237]. Shimabukuro observed that rat islets are also susceptible to FFA-induced cell death and found that ceramide was required for this process [238]. Low density lipoprotein (LDL) induces rat islet β cell death following cellular uptake and oxidation [239]. El-Assad and colleagues found that FFA-induced β-cell death could be increased with elevated glucose concentrations, again suggesting a role of oxidative stress in this cell death mechanism [240]. Primary and immortalized rat β cells were again shown to be sensitive to FFA-mediated cell death by Kharroubi et al. in 2004, and this work concluded that the cell death mechanism relied on an NF-kB and NO-independent form of ER stress [241]. Similarly, a mouse β-cell line exposed to palmitate exhibited cell death associated with changes in Ca^2+^ dynamics and increased CHOP expression, again suggesting ER stress-induced cell death [242]. Although clear evidence for FFA-induced β-cell death exists in the literature, it has been questioned whether the concentrations of FFAs used to induce β cell death in vitro are applicable to the in vivo setting of diabetes [78].

## 4. Potential Future Advances in Understanding β-Cell Death in Diabetes

As reviewed here, strong evidence exists to support the role of β-cell death in the insulin insufficiency and hyperglycemia that define diabetes. Significant advances have been made in understanding molecular mechanisms that can elicit β-cell death in vitro and in vivo. Why then, one asks, have these advances in knowledge not led to therapeutics that prevent β-cell loss in diabetes? Although several factors have contributed to this outcome, we suggest that new perspectives on the causes, mechanisms, and consequences of β-cell death are needed. It is recognized that a number of programmed cell death pathways operate in coordination in our cells, and that the functions of these mechanisms are not limited to death signaling [243]. Thus, understanding how and why these mechanisms of cell death exist may help us to understand β-cell death in the pathogenesis of diabetes [243]. Expanding our understanding of β-cell death in this way may lead to new strategies to protect β cells in the face of cytotoxic stress and prevent or cure diabetes.

### 4.1. Evidence for β-Cell Apoptosis, Necrosis, and Regulated Necrosis

#### 4.1.1. β-Cell Apoptosis

Apoptosis is a programed form of cell death that is required for development, normal cellular turnover, and tumor suppression [244,245]. The loss of β-cell mass in the setting of both T1D and T2D has thus far been attributed predominately to apoptosis [37,38,60,246]. Islets from individuals with type 2 diabetes show elevated markers of apoptosis, such as caspase-3 and caspase-8 [247]. The presence of certain morphological changes, such as cell shrinking, nuclear condensation, DNA fragmentation, exposure of phosphatidylserine on the plasma membrane, and membrane blebbing, can also be used to identify apoptosis [248,249]. Membrane blebs (also known as apoptotic bodies) encompass cellular content from apoptotic cells for phagocytosis by immune cells, thereby preventing release into the extracellular space and avoiding unwanted immune responses to this physiological process [250]. Thus, while apoptosis likely contributes to the loss of β cells in diabetes, it can also be regarded as a normal physiological process that is immunologically silent [251].

**Figure 2:**
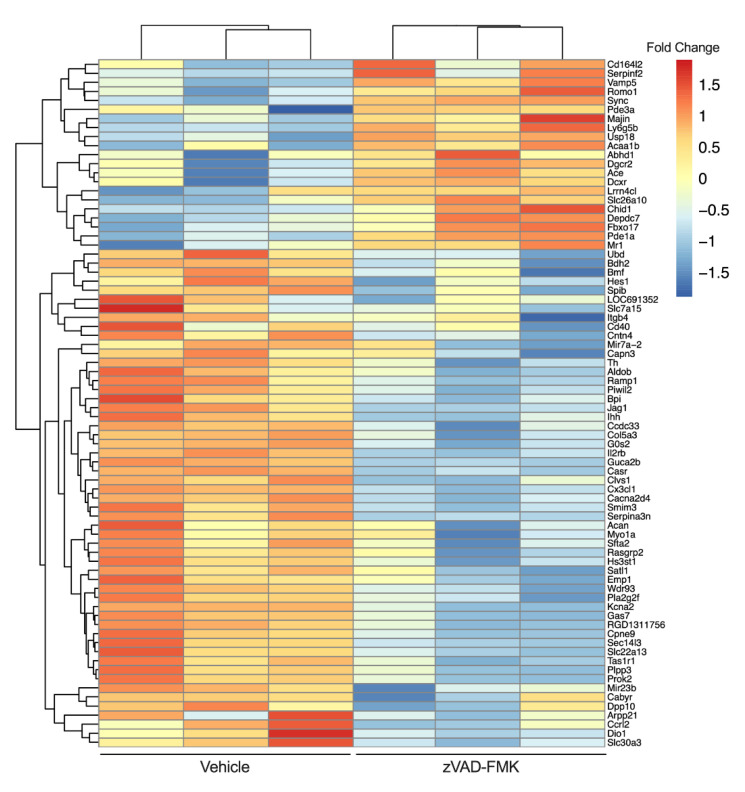
Inhibition of caspase activity in INS-1 832/13 β cells leads to differential gene expression. INS-1 832/13 β cells were treated with vehicle or the chemical pan-caspase inhibitor zVAD-FMK (50 μM) for 24 h in the presence of 11.1 mM glucose and subjected to RNAseq. Genes with the highest differential expression vs. mean, and with *p*-value and false discovery rate < 0.05, are displayed in the heatmap. n = 3 per condition.

Recently, we observed that the treatment of INS-1 832/13 β cells with a chemical pan-caspase inhibitor (zVAD-FMK) reduces caspase 3/7 activity to levels lower than those in vehicle treated cells [252]. This suggests that a basal level of caspase activity is present in β cells even in the absence of a death stimulus. To examine this physiological cysteine-aspartic protease activity, we performed RNAseq on vehicle and zVAD-FMK treated INS-1 β cells (Figure 2). We found that inhibition of caspase activity for 24 h resulted in upregulation of 3020 genes (including MHC class I-related (Mr1) and lymphocyte antigen 6 family member G5B (Ly6g5b)) and downregulation of 5146 genes (including TNF-receptor superfamily member 5 (Tnfrsf5, Cd40) and C-X3-C motif chemokine ligand 1 (Cx3cl1)). Thus, a basal level of caspase activity exists in β cells in the absence of death stimuli and may play a role in regulating the dialog with the immune system.

#### 4.1.2. β-Cell Necrosis

Necrosis is a lytic and immunogenic form of cell death traditionally regarded as unprogrammed and accidental, occurring, for example, in response to certain cellular trauma [253,254]. A study of BB rat islet cell death found necrosis to be the primary mode of cell death both in vitro and in vivo [255]. Steer et al. observed that IL-1β-mediated β-cell death was associated with HMGB1 release, but not caspase 3 activation or phosphatidylserine externalization, suggesting a lytic form of cell death, such as necrosis [256]. Collier and colleagues found that IL-1β and IFNγ treatment induced cell death with different caspase activation, mass spectrometry, and apoptosome signatures than were elicited by the apoptotic stimuli camptothecin or staurosporin [162]. These and other works suggest that β cells are susceptible to necrosis occurring downstream of cytokine signaling [257,258].

#### 4.1.3. β-Cell Regulated Necrosis

It has become appreciated recently that necrotic cell death is not always accidental and can instead occur downstream of regulated signaling events. Such types of regulated necrosis are lytic forms of cell death that can drive inflammation and immune responses following plasma membrane rupture and the release of cellular cargo into the extracellular environment [250]. Given previous observations of the molecular mechanisms of β-cell death, ferroptosis and necroptosis appear to be two forms of regulated necrosis with potential relevance to diabetes pathogenesis [252,259,260,261].

Ferroptosis is a form of regulated necrosis that is iron-dependent and characterized by lipid peroxidation that occurs due to insufficient redox enzyme activity, particularly glutathione peroxidase 4 (GPX4) [260,262]. A recent study revealed that the treatment of human islets with the ferroptosis-inducing agent erastin leads to a reduction of islet cell viability, and that this could be prevented by cotreatment with an iron chelator [259]. Given the low antioxidant content of β cells and their propensity for oxidative stress, one might anticipate they are susceptible to ferroptosis. Interestingly, individuals with elevated serum ferritin are at higher risk for metabolic syndrome [263], suggesting a possible role for ferroptosis in T2D associated β-cell loss.

Necroptosis is a form of regulated necrosis that occurs downstream of TNFR1 signaling, in the absence of caspase activation, and is mediated by receptor-interacting protein kinase 3 (RIPK3) and mixed lineage kinase domain-like pseudokinase (MLKL), molecules that have been identified in β cells [261,264,265,266]. Necroptosis results in lytic rupture of the cell membrane and release of damage-associated molecular patterns (DAMPs) that trigger immune responses [267]. This process is thought to be an evolutionarily conserved defense against caspase-inhibitory viral infections. Since T1D onset has been associated with viral infection [53,268,269], a potential link between β-cell necroptosis and T1D exists. In vitro studies have recently provided evidence that TNFα can elicit β-cell death when caspases are inhibited [252]. As discussed previously, the administration of a TNFα monoclonal antibody delays the β-cell functional decline in individuals with recently diagnosed T1D [175].

### 4.2. Differentiating between Forms of β-Cell Death

Differentiating between forms of cell death has challenged researchers since methods for identifying cell death became available. Necrosis had been identified as a mode of cell death by the 1960s [270,271], and Kerr, Wyllie, and Currie described apoptosis in 1972 [272]. In 1993, Buja et al. described these two forms of cell death, noting that apoptosis was characterized by endonuclease activity, and necrosis was associated with membrane damage [244]. In 1995, Majno and Joris applied additional terminology, such as programmed cell death, accidental cell death, and autolysis, to describe varying forms of cell death [273]. Until fairly recently, many have used the terms programmed cell death and apoptosis synonymously; that is, apoptosis was regarded as the only form of programmed cell death [274,275]. Examples of this are present throughout the β-cell death literature, and cell death is often called apoptosis despite a lack of direct evidence for apoptosis-specific signatures, such as caspase activation or presence of apoptotic bodies. Adding to this problem, several forms of programed necrosis have been described [250,276,277,278], so death that results from a regulated signaling event is not necessarily apoptosis.

Along these lines, many membrane-impermeable DNA-binding dyes used to detect cell death are marketed as “apoptosis” detection kits, although they are not specific for apoptotic cells versus cells undergoing other cell death processes. For example, terminal deoxynucleotidyl transferase dUTP nick end labeling (TUNEL) is often sold as an apoptosis specific dye, and it is commonly used in the literature to identify apoptotic cells [37,38,60]. However, TUNEL staining identifies both apoptotic and necrotic cells [279, 280]. Other DNA binding dyes used to detect cell death, such as propidium iodide (PI), have a similar lack of specificity [280,281].

As biochemical differences in these cell death pathways were identified, opportunities to distinguish between them arose. In 2009, a group of leading cell death researchers published “Classification of cell death: recommendations of the Nomenclature Committee on Cell Death” [282], and “Guidelines for the use and interpretation of assays for monitoring cell death in higher eukaryotes” [283]. Given the importance of understanding mechanisms of cell death in biomedical research, the authors encouraged more specific and accurate descriptions of the biochemical events associated with observations of cell death, and proposed methods to standardize identification of cell death types as well as terminology to use in this process. This leads one to ask, are β cells susceptible to non-apoptotic forms of cell death? If so, what might the importance of this be in the context of diabetes pathogenesis?

In addition to those discussed previously, several technologies are improving our ability both to monitor cell death and differentiate between various mechanisms of it. Identification of β-cell death in the context of diabetes has depended largely on measurements made in post-mortem pancreas sections or cells treated with cell death stimuli in vitro. A disadvantage of these approaches is that they are static and only provide data from a single time point. Recent developments in live-cell imaging and image analysis capabilities have enabled real-time monitoring of cell death over a period of days following treatment with death stimuli in vitro [284]. Such techniques add much needed resolution to quantifying the magnitude and temporal nature of mechanisms of cell death.

Advances that allow more accurate assessment of mechanisms of cell death have also been made. For example, several substrates that emit luminescent or fluorescent signals following cleavage by active caspases have been developed to monitor caspases in vitro, and these are amenable to both static and real-time assays [285]. Genetically encoded caspase activity biosensors have also been developed for use in cell-based assays [286]. Resources to monitor caspase activity in vivo have also been developed, including a mouse model using a LacZ reporter activated by caspase cleavage [287] and a fly model that allows differentiation between past and current caspase activity [288]. In contrast to these apoptosis specific measurements, Murai and colleagues recently developed a fluorescence resonance energy transfer (FRET) biosensor to monitor the interaction of RIPK3 and MLKL, and specifically identify necroptosis [289]. Tools such as these have provided new capabilities to interrogate mechanisms of cell death in more depth.

## 5. Conclusions

β-cell death is an important contributor to β-cell loss, insulin insufficiency, and hyperglycemia in both major forms of diabetes mellitus. We believe mechanisms that underlie cell death are not merely pathological, but may have important physiological roles as well. To prevent β-cell death in the pathogenesis of diabetes, we must better understand the evolutionarily conserved physiological functions of these mechanisms, and how they go awry in the pathophysiology of disease. Greater knowledge in these areas may lead to novel approaches to prevent β-cell death and maintain functional β-cell mass in diabetes.

## Data Availability

Data supporting the conclusions of this review are available in the literature cited in this paper and from the authors upon reasonable request.

## References

[1] Cho N.H., Shaw J.E., Karuranga S., Huang Y., da Rocha Fernandes J.D., Ohlrogge A.W., Malanda B. (2018). IDF Diabetes Atlas: Global estimates of diabetes prevalence for 2017 and projections for 2045. Diabetes Res. Clin. Pract..

[2] Beard H.A., Al Ghatrif M., Samper-Ternent R., Gerst K., Markides K.S. (2009). Trends in Diabetes Prevalence and Diabetes-Related Complications in Older Mexican Americans From 1993–1994 to 2004. Diabetes Care.

[3] American Diabetes Association (2020). Introduction: Standards of Medical Care in Diabetes. Diabetes Care.

[4] Kahn S.E. (2003). The relative contributions of insulin resistance and beta-cell dysfunction to the pathophysiology of Type 2 diabetes. Diabetologia.

[5] Klöppel G., Löhr M., Habich K., Oberholzer M., Heitz P.U. (1985). Islet Pathology and the Pathogenesis of Type 1 and Type 2 Diabetes mellitus Revisited. Surv. Synth. Pathol. Res..

[6] Cnop M., Welsh N., Jonas J.-C., Jörns A., Lenzen S., Eizirik D.L. (2005). Mechanisms of pancreatic beta-cell death in type 1 and type 2 diabetes: Many differences, few similarities. Diabetes.

[7] American Diabetes Association (2021). Classification and Diagnosis of Diabetes: Standards of Medical Care in Diabetes. Diabetes Care.

[8] Frank L.L. (1957). Diabetes mellitus in the texts of old Hindu medicine (Charaka, Susruta, Vagbhata). Am. J. Gastroenterol..

[9] Mering J.V., Minkowski O. (1890). Diabetes mellitus nach Pankreasexstirpation. Arch. Exp. Pathol. Pharmakol..

[10] Langerhans P.H. Contributions to the Microscopic Anatomy of the Pancreas. The Johns Hopkins University Press. https://www.jstor.org/stable/44438182.

[11] Opie E.L. (1901). The relation of diabetes mellitus to lesions of the pancreas. Hyaline degeneration of the islands of Langerhans. J. Exp. Med..

[12] Lane M.A. (1907). The cytological characters of the areas of Langerhans. Am. J. Anat..

[13] Banting F.G., Best C. (1963). The Internal Secretion of the Pancreas. Am. J. Physiol..

[14] Banting F.G., Best C.H., Collip J.B., Campbell W.R., Fletcher A.A., Macleod J.J.R., Noble E.C. (1922). The effect produced on diabetes by extractions of pancreas. Trans. Amer Physicians.

[15] Wellington A. (2020). Leonard Thompson ‘ever remembered’: The first person to receive insulin. J. Med. Biogr..

[16] Bliss M. (1982). The Discovery of Insulin: The Twenty-Fifth Anniversary Edition.

[17] Wrenshall G.A., Bogoch A., Ritchie R.C. (1952). Extractable Insulin of Pancreas: Correlation with Pathological and Clinical Findings in Diabetic and Nondiabetic Cases. Diabetes.

[18] MacLean N., Ogilvie R.F. (1955). Quantitative Estimation of the Pancreatic Islet Tissue in Diabetic Subjects. Diabetes.

[19] Von Engelhardt D., von Engelhardt D. (1989). Matthew Dobson (1735–1784). Clinical Investigator of Diabetes Mellitus. Diabetes Its Medical and Cultural History: Outlines—Texts—Bibliography.

[20] Steiner H. (1968). Insulitis beim perakuten Diabetes des Kindes. Klin. Wochenschr..

[21] Maccuish A., Irvine W., Barnes E., Duncan L. (1974). Antibodies to pancreatic islet cells in insulin-dependent diabetics with coexistent autoimmune disease. Lancet.

[22] Perley M.J., Kipnis D.M. (1967). Plasma Insulin Responses to Oral and Intravenous Glucose: Studies in Normal and Diabetic Subjects*. J. Clin. Investig..

[23] Bonadonna R.C., Leif G., Kraemer N., Ferrannini E., Del Prato S., DeFronzo R.A. (1990). Obesity and insulin resistance in humans: A dose-response study. Metabolism.

[24] MacLean N., Ogilvie R.F. (1959). Observations on the Pancreatic Islet Tissue of Young Diabetic Subjects. Diabetes.

[25] Doniach I., Morgan A.G. (1973). Islets of langerhans in juvenile diabetes mellitus. Clin. Endocrinol..

[26] Stefan Y., Orci L., Malaisse-Lagae F., Perrelet A., Patel Y., Unger R.H. (1982). Quantitation of Endocrine Cell Content in the Pancreas of Nondiabetic and Diabetic Humans. Diabetes.

[27] Drenck C.R., Oberholzer M., Heitz P.U. (1984). Morphometric evidence for a striking B-cell reduction at the clinical onset of type 1 diabetes. Virchows Arch. A.

[28] Johnson K.H., Stevens J.B. (1973). Light and Electron Microscopic Studies of Islet Amyloid in Diabetic Cats. Diabetes.

[29] Rahier J., Guiot Y., Goebbels R.M., Sempoux C., Henquin J.-C. (2008). Pancreatic β-cell mass in European subjects with type 2 diabetes. Diabetes Obes. Metab..

[30] MacCallum W.T. (1907). Hypertrophy of the islands of Langerhans in diabetes mellitus. Am. J. Med. Sci..

[31] Ogilvie R.F. (1933). The islands of langerhans in 19 cases of obesity. J. Pathol. Bacteriol..

[32] Prentki M. (2006). Islet cell failure in type 2 diabetes. J. Clin. Investig..

[33] Guiot Y., Sempoux C., Moulin P., Rahier J. (2001). No decrease of the beta-cell mass in type 2 diabetic patients. Diabetes.

[34] Cohrs C.M., Panzer J.K., Drotar D.M., Enos S.J., Kipke N., Chen C., Bozsak R., Schöniger E., Ehehalt F., Distler M. (2020). Dysfunction of Persisting β Cells Is a Key Feature of Early Type 2 Diabetes Pathogenesis. Cell Rep..

[35] Saito K., Yaginuma N., Takahashi T. (1979). Differential volumetry of A, B and D cells in the pancreatic islets of diabetic and nondiabetic subjects. Tohoku J. Exp. Med..

[36] Sakuraba H., Mizukami H., Yagihashi N., Wada R., Hanyu C. (2002). Reduced beta-cell mass and expression of oxidative stress-related DNA damage in the islet of Japanese Type II diabetic patients. Diabetologia.

[37] Butler A.E., Janson J., Bonner-Weir S., Ritzel R., Rizza R.A., Butler P.C. (2003). Beta-cell deficit and increased beta-cell apoptosis in humans with type 2 diabetes. Diabetes.

[38] Jurgens C.A., Toukatly M.N., Fligner C.L., Udayasankar J., Subramanian S.L., Zraika S., Aston-Mourney K., Carr D.B., Westermark P., Westermark G.T. (2011). β-Cell Loss and β-Cell Apoptosis in Human Type 2 Diabetes Are Related to Islet Amyloid Deposition. Am. J. Pathol..

[39] Wigger L., Barovic M., Brunner A.-D., Marzetta F., Schöniger E., Mehl F., Kipke N., Friedland D., Burdet F., Kessler C. (2021). Multi-omics profiling of living human pancreatic islet donors reveals heterogeneous beta cell trajectories towards type 2 diabetes. Nat. Metab..

[40] Redondo M.J., Hagopian W.A., Oram R., Steck A.K., Vehik K., Weedon M., Balasubramanyam A., Dabelea D. (2020). The clinical consequences of heterogeneity within and between different diabetes types. Diabetologia.

[41] Olehnik S.K., Fowler J.L., Avramovich G., Hara M. (2017). Quantitative analysis of intra- and inter-individual variability of human beta-cell mass. Sci. Rep..

[42] Ahlqvist E., Prasad R.B., Groop L. (2020). Subtypes of Type 2 Diabetes Determined From Clinical Parameters. Diabetes.

[43] Vendrame F., Zappaterreno A., Dotta F. (2004). Markers of beta cell function in type 1 diabetes mellitus. Minerva Med..

[44] Cerasi E., Luft R. (1967). The plasma insulin response to glucose infusion in healthy subjects and in diabetes mellitus. Eur. J. Endocrinol..

[45] Barker A., Lauria A., Schloot N., Hosszufalusi N., Ludvigsson J., Mathieu C., Mauricio D., Nordwall M., Van der Schueren B., Mandrup-Poulsen T. (2013). Age-dependent decline of β-cell function in type 1 diabetes after diagnosis: A multi-centre longitudinal study. Diabetes Obes. Metab..

[46] Yu M.G., Keenan H.A., Shah H.S., Frodsham S.G., Pober D., He Z., Wolfson E.A., D’Eon S., Tinsley L.J., Bonner-Weir S. (2019). Residual β cell function and monogenic variants in long-duration type 1 diabetes patients. J. Clin. Investig..

[47] Talchai C., Xuan S., Lin H., Sussel L., Accili D. (2012). Pancreatic β Cell Dedifferentiation as a Mechanism of Diabetic β Cell Failure. Cell.

[48] Rui J., Deng S., Arazi A., Perdigoto A.L., Liu Z., Herold K.C. (2017). β Cells that Resist Immunological Attack Develop during Progression of Autoimmune Diabetes in NOD Mice. Cell Metab..

[49] Karam J.H., Grodsky G.M., Forsham P.H., McWilliams N.B. (1963). Excessive Insulin Response to Glucose in Obese Subjects as Measured by Immunochemical Assay. Diabetes.

[50] Mitrakou A., Kelley D., Mokan M., Veneman T., Pangburn T., Reilly J., Gerich J. (1992). Role of Reduced Suppression of Glucose Production and Diminished Early Insulin Release in Impaired Glucose Tolerance. N. Engl. J. Med..

[51] Chen M., Bergman R.N., Pacini G., Porte D. (1985). Pathogenesis of Age-Related Glucose Intolerance in Man: Insulin Resistance and Decreased β-Cell Function*. J. Clin. Endocrinol. Metab..

[52] Utzschneider K.M., Prigeon R.L., Faulenbach M.V., Tong J., Carr D.B., Boyko E.J., Leonetti D.L., McNeely M.J., Fujimoto W.Y., Kahn S.E. (2008). Oral Disposition Index Predicts the Development of Future Diabetes Above and Beyond Fasting and 2-h Glucose Levels. Diabetes Care.

[53] Bottazzo G.F. (1986). Death of a Beta Cell: Homicide or Suicide?. Diabet. Med. J. Br. Diabet. Assoc..

[54] Kahn S.E. (2001). The Importance of β-Cell Failure in the Development and Progression of Type 2 Diabetes. J. Clin. Endocrinol. Metab..

[55] Gorczyca W., Bruno S., Darzynkiewicz R., Gong J. (1992). DNA strand breaks occurring during apoptosis—Their early insitu detection by the terminal deoxynucleotidyl transferase and nick translation assays and prevention by serine protease inhibitors. Int. J. Oncol..

[56] Vermes I., Haanen C., Steffens-Nakken H., Reutellingsperger C. (1995). A novel assay for apoptosis Flow cytometric detection of phosphatidylserine expression on early apoptotic cells using fluorescein labelled Annexin V. J. Immunol. Methods.

[57] Furuya T., Kamada T., Murakami T., Kurose A., Sasaki K. (1997). Laser scanning cytometry allows detection of cell death with morphological features of apoptosis in cells stained with PI. Cytometry.

[58] Meier J.J., Bhushan A., Butler A.E., Rizza R.A., Butler P.C. (2005). Sustained beta cell apoptosis in patients with long-standing type 1 diabetes: Indirect evidence for islet regeneration?. Diabetologia.

[59] Rhodes C.J. (2005). Type 2 Diabetes-a Matter of ß-Cell Life and Death?. Science.

[60] Butler A.E., Galasso R., Meier J.J., Basu R., Rizza R.A., Butler P.C. (2007). Modestly increased beta cell apoptosis but no increased beta cell replication in recent-onset type 1 diabetic patients who died of diabetic ketoacidosis. Diabetologia.

[61] Akirav E.M., Lebastchi J., Galvan E.M., Henegariu O., Akirav M., Ablamunits V., Lizardi P.M., Herold K.C. (2011). Detection of cell death in diabetes using differentially methylated circulating DNA. Proc. Natl. Acad. Sci. USA.

[62] Fisher M.M., Chumbiauca C.N.P., Mather K.J., Mirmira R.G., Tersey S.A. (2013). Detection of Islet β-Cell Death in Vivo by Multiplex PCR Analysis of Differentially Methylated DNA. Endocrinology.

[63] Husseiny M.I., Kaye A., Zebadua E., Kandeel F., Ferreri K. (2014). Tissue-Specific Methylation of Human Insulin Gene and PCR Assay for Monitoring Beta Cell Death. PLoS ONE.

[64] Lehmann-Werman R., Neiman D., Zemmour H., Moss J., Magenheim J., Vaknin-Dembinsky A., Rubertsson S., Nellgård B., Blennow K., Zetterberg H. (2016). Identification of tissue-specific cell death using methylation patterns of circulating DNA. Proc. Natl. Acad. Sci. USA.

[65] Neyman A., Nelson J., Tersey S.A., Mirmira R.G., Evans-Molina C., Sims E.K. (2018). Persistent elevations in circulating INS DNA among subjects with longstanding type 1 diabetes. Diabetes Obes. Metab..

[66] O’Brien B.A., Harmon B.V., Cameron D.P., Allan D.J. (1997). Apoptosis Is the Mode of -Cell Death Responsible for the Development of IDDM in the Nonobese Diabetic (NOD) Mouse. Diabetes.

[67] Augstein P., Elefanty A., Allison J., Harrison L.C. (1998). Apoptosis and beta-cell destruction in pancreatic islets of NOD mice with spontaneous and cyclophosphamide-accelerated diabetes. Diabetologia.

[68] Lally F.J., Ratcliff H., Bone A.J. (2001). Apoptosis and disease progression in the spontaneously diabetic BB/S rat. Diabetologia.

[69] Donath M.Y., Gross D.J., Cerasi E., Kaiser N. (1999). Hyperglycemia-induced beta-cell apoptosis in pancreatic islets of Psammomys obesus during development of diabetes. Diabetes.

[70] Pick A., Clark J., Kubstrup C., Levisetti M., Pugh W., Bonner-Weir S., Polonsky K.S. (1998). Role of apoptosis in failure of beta-cell mass compensation for insulin resistance and beta-cell defects in the male Zucker diabetic fatty rat. Diabetes.

[71] Farilla L., Hui H., Bertolotto C., Kang E., Bulotta A., Di Mario U., Perfetti R. (2002). Glucagon-Like Peptide-1 Promotes Islet Cell Growth and Inhibits Apoptosis in Zucker Diabetic Rats. Endocrinology.

[72] Donath M.Y., Halban P.A. (2004). Decreased beta-cell mass in diabetes: Significance, mechanisms and therapeutic implications. Diabetologia.

[73] Iwahashi H., Itoh N., Yamagata K., Imagawa A., Nakajima H., Tomita K., Moriwaki M., Waguri M., Yamamoto K., Miyagawa J. (1998). Molecular mechanisms of pancreatic beta-cell destruction in autoimmune diabetes: Potential targets for preventive therapy. Cytokines Cell. Mol. Ther..

[74] Hull R.L., Westermark G.T., Westermark P., Kahn S.E. (2004). Islet Amyloid: A Critical Entity in the Pathogenesis of Type 2 Diabetes. J. Clin. Endocrinol. Metab..

[75] Fonseca S.G., Gromada J., Urano F. (2011). Endoplasmic reticulum stress and pancreatic β-cell death. Trends Endocrinol. Metab..

[76] Drews G., Krippeit-Drews P., Düfer M. (2010). Oxidative stress and beta-cell dysfunction. Pflüg. Arch. Eur. J. Physiol..

[77] Lytrivi M., Castell A.-L., Poitout V., Cnop M. (2019). Recent Insights Into Mechanisms of β-Cell Lipo- and Glucolipotoxicity in Type 2 Diabetes. J. Mol. Biol..

[78] Weir G.C. (2019). Glucolipotoxicity, β-Cells, and Diabetes: The Emperor Has No Clothes. Diabetes.

[79] Coppieters K.T., Dotta F., Amirian N., Campbell P.D., Kay T.W., Atkinson M.A., Roep B.O., Von Herrath M.G. (2012). Demonstration of islet-autoreactive CD8 T cells in insulitic lesions from recent onset and long-term type 1 diabetes patients. J. Exp. Med..

[80] Babon J.A.B., DeNicola M.E., Blodgett D.M., Crèvecoeur I., Buttrick T.S., Maehr R., Bottino R., Naji A., Kaddis J., Elyaman W. (2016). Analysis of self-antigen specificity of islet-infiltrating T cells from human donors with type 1 diabetes. Nat. Med..

[81] Bottazzo G., Florin-Christensen A., Doniach D. (1974). Islet-cell antibodies in diabetes mellitus with autoimmune polyendocrine deficiencies. Lancet.

[82] Smeets S., De Paep D.L., Stangé G., Verhaeghen K., Van der Auwera B., Keymeulen B., Weets I., Ling Z., Veld P.I., Gorus F. (2021). Insulitis in the pancreas of non-diabetic organ donors under age 25 years with multiple circulating autoantibodies against islet cell antigens. Virchows Arch..

[83] Junker K., Egeberg J., Kromann H., Nerup J. (1977). An autopsy study of the islets of langerhans in acute-onset juvenile diabetes mellitus. Acta Pathol. Microbiol. Scand. Sect. A Pathol..

[84] Goel A., Chiu H., Felton J., Palmer J.P., Brooks-Worrell B. (2007). T-Cell Responses to Islet Antigens Improves Detection of Autoimmune Diabetes and Identifies Patients With More Severe β-Cell Lesions in Phenotypic Type 2 Diabetes. Diabetes.

[85] Herold K.C., Hagopian W., Auger J.A., Poumian-Ruiz E., Taylor L., Donaldson D., Gitelman S.E., Harlan D.M., Xu D., Zivin R.A. (2002). Anti-CD3 Monoclonal Antibody in New-Onset Type 1 Diabetes Mellitus. N. Engl. J. Med..

[86] Herold K.C., Gitelman S.E., Ehlers M.R., Gottlieb P.A., Greenbaum C.J., Hagopian W., Boyle K.D., Keyes-Elstein L., Aggarwal S., Phippard D. (2013). Teplizumab (Anti-CD3 mAb) Treatment Preserves C-Peptide Responses in Patients With New-Onset Type 1 Diabetes in a Randomized Controlled Trial: Metabolic and Immunologic Features at Baseline Identify a Subgroup of Responders. Diabetes.

[87] Demeester S., Keymeulen B., Kaufman L., Van Dalem A., Balti E.V., Van De Velde U., Goubert P., Verhaeghen K., Davidson H.W., Wenzlau J.M. (2015). Preexisting Insulin Autoantibodies Predict Efficacy of Otelixizumab in Preserving Residual β-Cell Function in Recent-Onset Type 1 Diabetes. Diabetes Care.

[88] Herold K.C., Bundy B.N., Long S.A., Bluestone J.A., DiMeglio L.A., Dufort M., Gitelman S.E., Gottlieb P.A., Krischer J.P., Linsley P.S. (2019). An Anti-CD3 Antibody, Teplizumab, in Relatives at Risk for Type 1 Diabetes. N. Engl. J. Med..

[89] Orban T., Bundy B., Becker D.J., DiMeglio L., Gitelman S.E., Goland R., Gottlieb P.A., Greenbaum C.J., Marks J.B., Monzavi R. (2011). Co-stimulation modulation with abatacept in patients with recent-onset type 1 diabetes: A randomised, double-blind, placebo-controlled trial. Lancet.

[90] Miyazaki A., Hanafusa T., Yamada K., Miyagawa J., Fujino-Kurihara H., Nakajima H., Nonaka K., Tarui S. (1985). Predominance of T lymphocytes in pancreatic islets and spleen of pre-diabetic non-obese diabetic (NOD) mice: A longitudinal study. Clin. Exp. Immunol..

[91] Christianson S.W., Shultz L.D., Leiter E.H. (1993). Adoptive Transfer of Diabetes Into Immunodeficient NOD-scid/scid Mice: Relative Contributions of CD4+ and CD8+ T-Cells From Diabetic Versus Prediabetic NOD.NON-Thy-1a Donors. Diabetes.

[92] Posselt A.M., Barker C.F., Friedman A.L., Naji A. (1992). Prevention of Autoimmune Diabetes in the BB Rat by Intrathymic Islet Transplantation at Birth. Science.

[93] Calderon B., Sacks D.B. (2014). Islet Autoantibodies and Type 1 Diabetes: Does the Evidence Support Screening?. Clin. Chem..

[94] Mullen Y. (2017). Development of the Nonobese Diabetic Mouse and Contribution of Animal Models for Understanding Type 1 Diabetes. Pancreas.

[95] Orban T., Sosenko J.M., Cuthbertson D., Krischer J.P., Skyler J.S., Jackson R., Yu L., Palmer J.P., Schatz D., Eisenbarth G. (2009). Pancreatic Islet Autoantibodies as Predictors of Type 1 Diabetes in the Diabetes Prevention Trial-Type. Diabetes Care.

[96] Pescovitz M.D., Greenbaum C.J., Bundy B., Becker D.J., Gitelman S.E., Goland R., Gottlieb P.A., Marks J.B., Moran A., Raskin P. (2013). B-Lymphocyte Depletion with Rituximab and—Cell Function: Two-Year Results. Diabetes Care.

[97] Mariño E., Silveira P.A., Stolp J., Grey S.T. (2011). B cell-directed therapies in type 1 diabetes. Trends Immunol..

[98] Zhang Y., O’Brien B., Trudeau J., Tan R., Santamaria P., Dutz J.P. (2002). In Situ β Cell Death Promotes Priming of Diabetogenic CD8 T Lymphocytes. J. Immunol..

[99] Tersey S.A., Nishiki Y., Templin A.T., Cabrera S.M., Stull N.D., Colvin S.C., Evans-Molina C., Rickus J.L., Maier B., Mirmira R.G. (2012). Islet -Cell Endoplasmic Reticulum Stress Precedes the Onset of Type 1 Diabetes in the Nonobese Diabetic Mouse Model. Diabetes.

[100] Engin F., Yermalovich A., Nguyen T., Hummasti S., Fu W., Eizirik D.L., Mathis D., Hotamisligil G.S. (2013). Restoration of the Unfolded Protein Response in Pancreatic β Cells Protects Mice Against Type 1 Diabetes. Sci. Transl. Med..

[101] Delong T., Wiles T.A., Baker R.L., Bradley B., Barbour G., Reisdorph R., Armstrong M., Powell R.L., Reisdorph N., Kumar N. (2016). Pathogenic CD4 T Cells in Type 1 Diabetes Recognize Epitopes Formed by Peptide Fusion. Science.

[102] Eizirik D.L., Colli M.L., Ortis F. (2009). The role of inflammation in insulitis and β-cell loss in type 1 diabetes. Nat. Rev. Endocrinol..

[103] Oram R.A., Sims E.K., Evans-Molina C. (2019). Beta cells in type 1 diabetes: Mass and function; sleeping or dead?. Diabetologia.

[104] Sims E.K., Mirmira R.G., Evans-Molina C. (2020). The role of beta-cell dysfunction in early type 1 diabetes. Curr. Opin. Endocrinol. Diabetes Obes..

[105] Roep B.O., Thomaidou S., van Tienhoven R., Zaldumbide A. (2020). Type 1 diabetes mellitus as a disease of the β-cell (do not blame the immune system?). Nat. Rev. Endocrinol..

[106] Westermark G.T., Krogvold L., Dahl-Jørgensen K., Ludvigsson J. (2017). Islet amyloid in recent-onset type 1 diabetes—The DiViD study. Upsala J. Med. Sci..

[107] Paulsson J.F., Ludvigsson J., Carlsson A., Casas R., Forsander G., Ivarsson S.A., Kockum I., Lernmark Å., Marcus C., Lindblad B. (2014). High Plasma Levels of Islet Amyloid Polypeptide in Young with New-Onset of Type 1 Diabetes Mellitus. PLoS ONE.

[108] Delong T., Baker R.L., Reisdorph N., Powell R.L., Armstrong M., Barbour G., Bradley B., Haskins K. (2011). Islet Amyloid Polypeptide Is a Target Antigen for Diabetogenic CD4+ T Cells. Diabetes.

[109] Westermark G.T., Westermark P., Berne C., Korsgren O., Nordic Network for Clinical Islet Transplantation (2008). Widespread Amyloid Deposition in Transplanted Human Pancreatic Islets. N. Engl. J. Med..

[110] Westermark G.T., Davalli A.M., Secchi A., Folli F., Kin T., Toso C., Shapiro A.M.J., Korsgren O., Tufveson G., Andersson A. (2012). Further Evidence for Amyloid Deposition in Clinical Pancreatic Islet Grafts. Transplantation.

[111] Westermark P., Wernstedt C., Wilander E., Hayden D.W., O’Brien T.D., Johnson K.H. (1987). Amyloid fibrils in human insulinoma and islets of Langerhans of the diabetic cat are derived from a neuropeptide-like protein also present in normal islet cells. Proc. Natl. Acad. Sci. USA.

[112] Kahn S.E., D’Alessio D.A., Schwartz M.W., Fujimoto W.Y., Ensinck J.W., Taborsky G.J., Porte D. (1990). Evidence of cosecretion of islet amyloid polypeptide and insulin by beta-cells. Diabetes.

[113] Scherbaum W.A. (1998). The role of amylin in the physiology of glycemic control. Exp. Clin. Endocrinol. Diabetes.

[114] Rushing P.A., Hagan M.M., Seeley R.J., Lutz T.A., Woods S.C. (2000). Amylin: A Novel Action in the Brain to Reduce Body Weight*. Endocrinology.

[115] Westermark P., Engstrom U., Johnson K.H., Westermark G.T., Betsholtz C. (1990). Islet amyloid polypeptide: Pinpointing amino acid residues linked to amyloid fibril formation. Proc. Natl. Acad. Sci. USA.

[116] Lorenzo A., Razzaboni B., Weir G.C., Yankner B.A. (1994). Pancreatic islet cell toxicity of amylin associated with type-2 diabetes mellitus. Nature.

[117] Lopes D.H., Colin C., Degaki T.L., de Sousa A.C.V., Vieira M.N., Sebollela A., Martinez A.M.B., Bloch C., Ferreira S.T., Sogayar M.C. (2004). Amyloidogenicity and Cytotoxicity of Recombinant Mature Human Islet Amyloid Polypeptide (rhIAPP). J. Biol. Chem..

[118] Tenidis K., Waldner M., Bernhagen J., Fischle W., Bergmann M., Weber M., Merkle M.-L., Voelter W., Brunner H., Kapurniotu A. (2000). Identification of a penta- and hexapeptide of islet amyloid polypeptide (IAPP) with amyloidogenic and cytotoxic properties. J. Mol. Biol..

[119] Meier J.J., Kayed R., Lin C.-Y., Gurlo T., Haataja L., Jayasinghe S., Langen R., Glabe C.G., Butler P.C. (2006). Inhibition of human IAPP fibril formation does not prevent β-cell death: Evidence for distinct actions of oligomers and fibrils of human IAPP. Am. J. Physiol. Metab..

[120] Bram Y., Frydman-Marom A., Yanai I., Gilead S., Shaltiel-Karyo R., Amdursky N., Gazit E. (2014). Apoptosis induced by islet amyloid polypeptide soluble oligomers is neutralized by diabetes-associated specific antibodies. Sci. Rep..

[121] Chen W.-J., Armour S., Way J., Chen G., Watson C., Irving P., Cobb J., Kadwell S., Beaumont K., Rimele T. (1997). Expression Cloning and Receptor Pharmacology of Human Calcitonin Receptors from MCF-7 Cells and Their Relationship to Amylin Receptors. Mol. Pharmacol..

[122] Lee S., Hay D., Pioszak A.A. (2016). Calcitonin and Amylin Receptor Peptide Interaction Mechanisms: Insights into peptide-binding modes and allosteric modulation of the calcitonin receptor by receptor activity-modifying proteins. J. Biol. Chem..

[123] Abedini A., Cao P., Plesner A., Zhang J., He M., Derk J., Patil S.A., Rosario R., Lonier J., Song F. (2018). RAGE binds preamyloid IAPP intermediates and mediates pancreatic β cell proteotoxicity. J. Clin. Investig..

[124] Bower R.L., Yule L., Rees T.A., Deganutti G., Hendrikse E.R., Harris P.W.R., Kowalczyk R., Ridgway Z., Wong A.G., Swierkula K. (2018). Molecular Signature for Receptor Engagement in the Metabolic Peptide Hormone Amylin. ACS Pharmacol. Transl. Sci..

[125] Mirzabekov T.A., Lin M.-C., Kagan B.L. (1996). Pore Formation by the Cytotoxic Islet Amyloid Peptide Amylin. J. Biol. Chem..

[126] Janson J., Ashley R.H., Harrison D., McIntyre S., Butler P. (1999). The mechanism of islet amyloid polypeptide toxicity is membrane disruption by intermediate-sized toxic amyloid particles. Diabetes.

[127] Cao P., Abedini A., Wang H., Tu L.-H., Zhang X., Schmidt A.M., Raleigh D.P. (2013). Islet amyloid polypeptide toxicity and membrane interactions. Proc. Natl. Acad. Sci. USA.

[128] Birol M., Kumar S., Rhoades E., Miranker A.D. (2018). Conformational switching within dynamic oligomers underpins toxic gain-of-function by diabetes-associated amyloid. Nat. Commun..

[129] Masters S.L., Dunne A., Subramanian S.L., Hull R.L., Tannahill G.M., Sharp F.A., Becker C., Franchi L., Yoshihara E., Chen Z. (2010). Activation of the NLRP3 inflammasome by islet amyloid polypeptide provides a mechanism for enhanced IL-1β in type 2 diabetes. Nat. Immunol..

[130] Westwell-Roper C.Y., Chehroudi C.A., Denroche H., Courtade J.A., Ehses J., Verchere C.B. (2014). IL-1 mediates amyloid-associated islet dysfunction and inflammation in human islet amyloid polypeptide transgenic mice. Diabetologia.

[131] Schlamadinger D.E., Miranker A.D. (2014). Fiber-Dependent and -Independent Toxicity of Islet Amyloid Polypeptide. Biophys. J..

[132] Hopping G., Kellock J., Barnwal R.P., Law P., Bryers J., Varani G., Caughey B., Daggett V. (2014). Designed α-sheet peptides inhibit amyloid formation by targeting toxic oligomers. eLife.

[133] Verchere C.B., D’Alessio D.A., Palmiter R.D., Weir G.C., Bonner-Weir S., Baskin D.G., Kahn S.E. (1996). Islet amyloid formation associated with hyperglycemia in transgenic mice with pancreatic beta cell expression of human islet amyloid polypeptide. Proc. Natl. Acad. Sci. USA.

[134] Janson J., Soeller W.C., Roche P.C., Nelson R.T., Torchia A.J., Kreutter D.K., Butler P.C. (1996). Spontaneous diabetes mellitus in transgenic mice expressing human islet amyloid polypeptide. Proc. Natl. Acad. Sci. USA.

[135] Soeller W.C., Janson J., Hart S.E., Parker J.C., Carty M.D., Stevenson R.W., Kreutter D.K., Butler P.C. (1998). Islet amyloid-associated diabetes in obese A(vy)/a mice expressing human islet amyloid polypeptide. Diabetes.

[136] Butler A.E., Jang J., Gurlo T., Carty M.D., Soeller W.C., Butler P.C. (2004). Diabetes due to a progressive defect in beta-cell mass in rats transgenic for human islet amyloid polypeptide (HIP Rat): A new model for type 2 diabetes. Diabetes.

[137] Andrikopoulos S., Verchere C.B., Terauchi Y., Kadowaki T., Kahn S.E. (2000). beta-cell glucokinase deficiency and hyperglycemia are associated with reduced islet amyloid deposition in a mouse model of type 2 diabetes. Diabetes.

[138] Zraika S., Hull R.L., Udayasankar J., Utzschneider K.M., Tong J., Gerchman F., Kahn S.E. (2007). Glucose- and time-dependence of islet amyloid formation in vitro. Biochem. Biophys. Res. Commun..

[139] Westwell-Roper C., Dai D.L., Soukhatcheva G., Potter K.J., Van Rooijen N., Ehses J.A., Verchere C.B. (2011). IL-1 Blockade Attenuates Islet Amyloid Polypeptide-Induced Proinflammatory Cytokine Release and Pancreatic Islet Graft Dysfunction. J. Immunol..

[140] Westwell-Roper C.Y., Ehses J.A., Verchere C.B. (2013). Resident Macrophages Mediate Islet Amyloid Polypeptide–Induced Islet IL-1β Production and β-Cell Dysfunction. Diabetes.

[141] Meier D.T., Morcos M., Samarasekera T., Zraika S., Hull R., Kahn S.E. (2014). Islet amyloid formation is an important determinant for inducing islet inflammation in high-fat-fed human IAPP transgenic mice. Diabetologia.

[142] Templin A.T., Mellati M., Meier D.T., Esser N., Hogan M.F., Castillo J.J., Akter R., Raleigh D.P., Zraika S., Hull R.L. (2020). Low concentration IL-1β promotes islet amyloid formation by increasing hIAPP release from humanised mouse islets in vitro. Diabetologia.

[143] Westwell-Roper C., Denroche H., Ehses J., Verchere C.B. (2016). Differential Activation of Innate Immune Pathways by Distinct Islet Amyloid Polypeptide (IAPP) Aggregates. J. Biol. Chem..

[144] Huang C.-J., Lin C.-Y., Haataja L., Gurlo T., Butler A.E., Rizza R.A., Butler P.C. (2007). High Expression Rates of Human Islet Amyloid Polypeptide Induce Endoplasmic Reticulum Stress–Mediated β-Cell Apoptosis, a Characteristic of Humans With Type 2 but Not Type 1 Diabetes. Diabetes.

[145] Gurlo T., Rivera J.F., Butler A.E., Cory M., Hoang J., Costes S., Butler P. (2016). CHOP Contributes to, But Is Not the Only Mediator of, IAPP Induced β-Cell Apoptosis. Mol. Endocrinol..

[146] Meng F., Abedini A., Plesner A., Middleton C.T., Potter K.J., Zanni M.T., Verchere C.B., Raleigh D.P. (2010). The Sulfated Triphenyl Methane Derivative Acid Fuchsin Is a Potent Inhibitor of Amyloid Formation by Human Islet Amyloid Polypeptide and Protects against the Toxic Effects of Amyloid Formation. J. Mol. Biol..

[147] Ren B., Liu Y., Zhang Y., Cai Y., Gong X., Chang Y., Xu L., Zheng J. (2018). Genistein: A Dual Inhibitor of Both Amyloid β and Human Islet Amylin Peptides. ACS Chem. Neurosci..

[148] Saravanan M.S., Ryazanov S., Leonov A., Nicolai J., Praest P., Giese A., Winter R., Khemtemourian L., Griesinger C., Killian J.A. (2019). The small molecule inhibitor anle145c thermodynamically traps human islet amyloid peptide in the form of non-cytotoxic oligomers. Sci. Rep..

[149] Oskarsson M.E., Hermansson E., Wang Y., Welsh N., Presto J., Johansson J., Westermark G.T. (2018). BRICHOS domain of Bri2 inhibits islet amyloid polypeptide (IAPP) fibril formation and toxicity in human beta cells. Proc. Natl. Acad. Sci. USA.

[150] Armiento V., Hille K., Naltsas D., Lin J.S., Barron A.E., Kapurniotu A. (2020). The Human Host-Defense Peptide Cathelicidin LL-37 is a Nanomolar Inhibitor of Amyloid Self-Assembly of Islet Amyloid Polypeptide (IAPP). Angew. Chem. Int. Ed..

[151] Röder C., Kupreichyk T., Gremer L., Schäfer L.U., Pothula K.R., Ravelli R.B.G., Willbold D., Hoyer W., Schröder G.F. (2020). Cryo-EM structure of islet amyloid polypeptide fibrils reveals similarities with amyloid-β fibrils. Nat. Struct. Mol. Biol..

[152] Roesti E.S., Boyle C.N., Zeman D.T., Sande-Melon M., Storni F., Cabral-Miranda G., Knuth A., Lutz T.A., Vogel M., Bachmann M.F. (2020). Vaccination Against Amyloidogenic Aggregates in Pancreatic Islets Prevents Development of Type 2 Diabetes Mellitus. Vaccines.

[153] Lin C.-Y., Gurlo T., Kayed R., Butler A.E., Haataja L., Glabe C.G., Butler P.C. (2007). Toxic Human Islet Amyloid Polypeptide (h-IAPP) Oligomers Are Intracellular, and Vaccination to Induce Anti-Toxic Oligomer Antibodies Does Not Prevent h-IAPP-Induced -Cell Apoptosis in h-IAPP Transgenic Mice. Diabetes.

[154] Palmer J.P., Helqvist S., Spinas G.A., Mølvig J., Mandrup-Poulsen T., Andersen H.U., Nerup J. (1989). Interaction of -Cell Activity and IL-1 Concentration and Exposure Time in Isolated Rat Islets of Langerhans. Diabetes.

[155] Dror E., Dalmas E., Zeman-Meier D., Wueest S., Thévenet J., Thienel C., Timper K., Nordmann T.M., Traub S., Schulze F. (2017). Postprandial macrophage-derived IL-1β stimulates insulin, and both synergistically promote glucose disposal and inflammation. Nat. Immunol..

[156] Cardozo A.K., Ortis F., Storling J., Feng Y.-M., Rasschaert J., Tonnesen M., Van Eylen F., Mandrup-Poulsen T., Herchuelz A., Eizirik D.L. (2005). Cytokines downregulate the sarcoendoplasmic reticulum pump Ca^2+^ ATPase 2b and deplete endoplasmic reticulum Ca2+, leading to induction of endoplasmic reticulum stress in pancreatic beta-cells. Diabetes.

[157] Corbett J.A., Wang J.L., Misko T.P., Zhao W., Hickey W.F., Mcdaniel M.L. (1993). Nitric Oxide Mediates IL-1β-Induced Islet Dysfunction and Destruction: Prevention by Dexamethasone. Autoimmunity.

[158] Chambers K.T., Unverferth J.A., Weber S.M., Wek R.C., Urano F., Corbett J.A. (2007). The Role of Nitric Oxide and the Unfolded Protein Response in Cytokine-Induced -Cell Death. Diabetes.

[159] Hughes K.J., Chambers K.T., Meares G.P., Corbett J.A. (2009). Nitric oxides mediates a shift from early necrosis to late apoptosis in cytokine-treated β-cells that is associated with irreversible DNA damage. Am. J. Physiol. Endocrinol. Metab..

[160] D’Hertog W., Overbergh L., Lage K., Ferreira G.B., Maris M., Gysemans C., Flamez D., Cardozo A.K., Bergh G.V.D., Schoofs L. (2007). Proteomics Analysis of Cytokine-induced Dysfunction and Death in Insulin-producing INS-1E Cells: New insights into the pathways involved. Mol. Cell. Proteom..

[161] Ehses J., Böni-Schnetzler M., Faulenbach M., Donath M.Y. (2008). Macrophages, cytokines and β-cell death in Type 2 diabetes. Biochem. Soc. Trans..

[162] Collier J.J., Burke S.J., Eisenhauer M.E., Lu D., Sapp R.C., Frydman C.J., Campagna S. (2011). Pancreatic β-Cell Death in Response to Pro-Inflammatory Cytokines Is Distinct from Genuine Apoptosis. PLoS ONE.

[163] Demine S., Schiavo A.A., Marín-Cañas S., Marchetti P., Cnop M., Eizirik D.L. (2020). Pro-inflammatory cytokines induce cell death, inflammatory responses, and endoplasmic reticulum stress in human iPSC-derived beta cells. Stem Cell Res. Ther..

[164] Corbett J.A., Wang J.L., Sweetland M.A., Jr J.R.L., McDaniel M.L. Interleukin 1 Beta Induces the Formation of Nitric Oxide by Beta-Cells Purified from Rodent Islets of Langerhans. Evidence for the Beta-Cell as a Source and Site of Action of Nitric Oxide. https://www.jci.org/articles/view/116129/scanned-page/2384.

[165] Ammendrup A., Maillard A., Nielsen K., Andersen N.A., Serup P., Madsen O.D., Mandrup-Poulsen T., Bonny C. (2000). The c-Jun amino-terminal kinase pathway is preferentially activated by interleukin-1 and controls apoptosis in differentiating pancreatic beta-cells. Diabetes.

[166] Wang Q., Zhang H., Zhao B., Fei H. (2009). IL-1β caused pancreatic β-cells apoptosis is mediated in part by endoplasmic reticulum stress via the induction of endoplasmic reticulum Ca2+ release through the c-Jun N-terminal kinase pathway. Mol. Cell. Biochem..

[167] Schwarznau A., Hanson M.S., Sperger J.M., Schram B.R., Danobeitia J.S., Greenwood K.K., Vijayan A., Fernandez L.A. (2009). IL-1β receptor blockade protects islets against pro-inflammatory cytokine induced necrosis and apoptosis. J. Cell. Physiol..

[168] Dupraz P., Cottet S., Hamburger F., Dolci W., Felley-Bosco E., Thorens B. (2000). Dominant Negative MyD88 Proteins Inhibit Interleukin-1β/Interferon-γ-mediated Induction of Nuclear Factor κB-dependent Nitrite Production and Apoptosis in β Cells. J. Biol. Chem..

[169] Carswell E.A., Old L.J., Kassel R.L., Green S., Fiore N., Williamson B. (1975). An endotoxin-induced serum factor that causes necrosis of tumors. Proc. Natl. Acad. Sci. USA.

[170] Decker T., Lohmann-Matthes M.L., Gifford G.E. (1987). Cell-associated tumor necrosis factor (TNF) as a killing mechanism of activated cytotoxic macrophages. J. Immunol..

[171] Ishizuka N., Yagui K., Tokuyama Y., Yamada K., Suzuki Y., Miyazaki J.-I., Hashimoto N., Makino H., Saito Y., Kanatsuka A. (1999). Tumor necrosis factor alpha signaling pathway and apoptosis in pancreatic β cells. Metabolism.

[172] Green E., Eynon E.E., Flavell R.A. (1998). Local Expression of TNFα in Neonatal NOD Mice Promotes Diabetes by Enhancing Presentation of Islet Antigens. Immunity.

[173] Kägi D., Ho A., Odermatt B., Zakarian A., Ohashi P.S., Mak T.W. (1999). TNF receptor 1-dependent beta cell toxicity as an effector pathway in autoimmune diabetes. J. Immunol. Baltim. Md. 1950.

[174] Stephens L.A., Thomas H.E., Ming L., Darwiche M.G.R., Volodin L., Kay T.W.H. (1999). Tumor Necrosis Factor-α-Activated Cell Death Pathways in NIT-1 Insulinoma Cells and Primary Pancreatic β Cells*. Endocrinology.

[175] Quattrin T., Haller M.J., Steck A.K., Felner E.I., Li Y., Xia Y., Leu J.H., Zoka R., Hedrick J.A., Rigby M.R. (2020). Golimumab and Beta-Cell Function in Youth with New-Onset Type 1 Diabetes. N. Engl. J. Med..

[176] Campbell I.L., Wong G.H.W., Schrader J.W., Harrison L.C. (1985). Interferon- Enhances the Expression of the Major Histocompatibility Class I Antigens on Mouse Pancreatic Beta Cells. Diabetes.

[177] Campbell I.L., Oxbrow L., Harrison L.C. (1987). Interferon-γ: Pleiotropic effects on a rat pancreatic beta cell line. Mol. Cell. Endocrinol..

[178] Thomas H.E., Parker J.L., Schreiber R.D., Kay T.W. (1998). IFN-gamma action on pancreatic beta cells causes class I MHC upregulation but not diabetes. J. Clin. Investig..

[179] Ferreira R.C., Guo H., Coulson R.M., Smyth D.J., Pekalski M.L., Burren O.S., Cutler A.J., Doecke J.D., Flint S., McKinney E.F. (2014). A Type I Interferon Transcriptional Signature Precedes Autoimmunity in Children Genetically at Risk for Type 1 Diabetes. Diabetes.

[180] Marroqui L., dos Santos R.S., De Beeck A.O., De Brachène A.C., Marselli L., Marchetti P., Eizirik D.L. (2017). Interferon-α mediates human beta cell HLA class I overexpression, endoplasmic reticulum stress and apoptosis, three hallmarks of early human type 1 diabetes. Diabetologia.

[181] Mori H., Takahashi H., Mine K., Higashimoto K., Inoue K., Kojima M., Kuroki S., Eguchi T., Ono Y., Inuzuka S. (2021). *TYK2* Promoter Variant Is Associated with Impaired Insulin Secretion and Lower Insulin Resistance in Japanese Type 2 Diabetes Patients. Genes.

[182] Izumi K., Mine K., Inoue Y., Teshima M., Ogawa S., Kai Y., Kurafuji T., Hirakawa K., Miyakawa D., Ikeda H. (2015). Reduced Tyk2 gene expression in β-cells due to natural mutation determines susceptibility to virus-induced diabetes. Nat. Commun..

[183] Marroqui L., Dos Santos R.S., Fløyel T., Grieco F.A., Santin I., de Beeck A.O., Marselli L., Marchetti P., Pociot F., Eizirik D.L. (2015). TYK2, a Candidate Gene for Type 1 Diabetes, Modulates Apoptosis and the Innate Immune Response in Human Pancreatic β-Cells. Diabetes.

[184] Eizirik D.L., Cardozo A.K., Cnop M. (2007). The Role for Endoplasmic Reticulum Stress in Diabetes Mellitus. Endocr. Rev..

[185] Ma Y., Hendershot L.M. (2003). Delineation of a Negative Feedback Regulatory Loop That Controls Protein Translation during Endoplasmic Reticulum Stress. J. Biol. Chem..

[186] Back S.H., Scheuner D., Han J., Song B., Ribick M., Wang J., Gildersleeve R.D., Pennathur S., Kaufman R.J. (2009). Translation Attenuation through eIF2α Phosphorylation Prevents Oxidative Stress and Maintains the Differentiated State in β Cells. Cell Metab..

[187] Ron D. (2002). Proteotoxicity in the endoplasmic reticulum: Lessons from the Akita diabetic mouse. J. Clin. Investig..

[188] Hara T., Mahadevan J., Kanekura K., Hara M., Lu S., Urano F. (2014). Calcium Efflux From the Endoplasmic Reticulum Leads to β-Cell Death. Endocrinology.

[189] Kono T., Tong X., Taleb S., Bone R.N., Iida H., Lee C.-C., Sohn P., Gilon P., Roe M.W., Evans-Molina C. (2018). Impaired Store-Operated Calcium Entry and STIM1 Loss Lead to Reduced Insulin Secretion and Increased Endoplasmic Reticulum Stress in the Diabetic β-Cell. Diabetes.

[190] Ozcan U., Yilmaz E., Ozcan L., Furuhashi M., Vaillancourt E., Smith R.O., Görgün C.Z., Hotamisligil G.S. (2006). Chemical chaperones reduce ER stress and restore glucose homeostasis in a mouse model of type 2 diabetes. Science.

[191] Tang C., Koulajian K., Schuiki I., Zhang L., Desai T., Ivovic A., Wang P., Robson-Doucette C., Wheeler M.B., Minassian B. (2012). Glucose-induced beta cell dysfunction in vivo in rats: Link between oxidative stress and endoplasmic reticulum stress. Diabetologia.

[192] Rao R.V., Ellerby H.M., Bredesen D.E. (2004). Coupling endoplasmic reticulum stress to the cell death program. Cell Death Differ..

[193] O’Sullivan-Murphy B., Urano F. (2012). ER Stress as a Trigger for -Cell Dysfunction and Autoimmunity in Type 1 Diabetes. Diabetes.

[194] Holcik M., Sonenberg N. (2005). Translational control in stress and apoptosis. Nat. Rev. Mol. Cell Biol..

[195] Gardner B.M., Pincus D., Gotthardt K., Gallagher C.M., Walter P. (2013). Endoplasmic Reticulum Stress Sensing in the Unfolded Protein Response. Cold Spring Harb. Perspect. Biol..

[196] Laybutt D.R., Preston A.M., Åkerfeldt M.C., Kench J., Busch A.K., Biankin A., Biden T.J. (2007). Endoplasmic reticulum stress contributes to beta cell apoptosis in type 2 diabetes. Diabetologia.

[197] Marchetti P., Bugliani M., Lupi R., Marselli L., Masini M., Boggi U., Filipponi F., Weir G.C., Eizirik D.L., Cnop M. (2007). The endoplasmic reticulum in pancreatic beta cells of type 2 diabetes patients. Diabetologia.

[198] Nakatani Y., Kaneto H., Kawamori D., Yoshiuchi K., Hatazaki M., Matsuoka T.-A., Ozawa K., Ogawa S., Hori M., Yamasaki Y. (2005). Involvement of Endoplasmic Reticulum Stress in Insulin Resistance and Diabetes. J. Biol. Chem..

[199] Zinszner H., Kuroda M., Wang X., Batchvarova N., Lightfoot R.T., Remotti H., Stevens J.L., Ron D. (1998). CHOP is implicated in programmed cell death in response to impaired function of the endoplasmic reticulum. Genes Dev..

[200] Marciniak S., Yun C.Y., Oyadomari S., Novoa I., Zhang Y., Jungreis R., Nagata K., Harding H., Ron D. (2004). CHOP induces death by promoting protein synthesis and oxidation in the stressed endoplasmic reticulum. Genes Dev..

[201] Oyadomari S., Koizumi A., Takeda K., Gotoh T., Akira S., Araki E., Mori M. (2002). Targeted disruption of the Chop gene delays endoplasmic reticulum stress–mediated diabetes. J. Clin. Investig..

[202] Song B., Scheuner D., Ron D., Pennathur S., Kaufman R.J. (2008). Chop deletion reduces oxidative stress, improves β cell function, and promotes cell survival in multiple mouse models of diabetes. J. Clin. Investig..

[203] Mahajan A., Taliun D., Thurner M., Robertson N.R., Torres J.M., Rayner N.W., Payne A.J., Steinthorsdottir V., Scott R.A., Grarup N. (2018). Fine-mapping type 2 diabetes loci to single-variant resolution using high-density imputation and islet-specific epigenome maps. Nat. Genet..

[204] Vujkovic M., Keaton J.M., Lynch J.A., Miller D.R., Zhou J., Tcheandjieu C., Huffman J.E., Assimes T.L., Lorenz K., Zhu X. (2020). Discovery of 318 new risk loci for type 2 diabetes and related vascular outcomes among 1.4 million participants in a multi-ancestry meta-analysis. Nat. Genet..

[205] Abreu D., Asada R., Revilla J.M.P., Lavagnino Z., Kries K., Piston D.W., Urano F. (2020). Wolfram syndrome 1 gene regulates pathways maintaining beta-cell health and survival. Lab. Investig. J. Tech. Methods Pathol..

[206] Yang C., Diiorio P., Jurczyk A., O’Sullivan-Murphy B., Urano F., Bortell R. (2013). Pathological endoplasmic reticulum stress mediated by the IRE1 pathway contributes to pre-insulitic beta cell apoptosis in a virus-induced rat model of type 1 diabetes. Diabetologia.

[207] Lee H., Lee Y.-S., Harenda Q., Pietrzak S., Oktay H.Z., Schreiber S., Liao Y., Sonthalia S., Ciecko A.E., Chen Y.-G. (2020). Beta Cell Dedifferentiation Induced by IRE1α Deletion Prevents Type 1 Diabetes. Cell Metab..

[208] Morita S., Villalta S.A., Feldman H.C., Register A.C., Rosenthal W., Hoffmann-Petersen I.T., Mehdizadeh M., Ghosh R., Wang L., Colon-Negron K. (2017). Targeting ABL-IRE1α Signaling Spares ER-Stressed Pancreatic β Cells to Reverse Autoimmune Diabetes. Cell Metab..

[209] Delépine M., Nicolino M., Barrett T., Golamaully M., Lathrop G.M., Julier C. (2000). EIF2AK3, encoding translation initiation factor 2-α kinase 3, is mutated in patients with Wolcott-Rallison syndrome. Nat. Genet..

[210] Stone S.I., Abreu D., McGill J.B., Urano F. (2020). Monogenic and syndromic diabetes due to endoplasmic reticulum stress. J. Diabetes Its Complicat..

[211] Lu S., Kanekura K., Hara T., Mahadevan J., Spears L.D., Oslowski C.M., Martinez R., Yamazaki-Inoue M., Toyoda M., Neilson A. (2014). A calcium-dependent protease as a potential therapeutic target for Wolfram syndrome. Proc. Natl. Acad. Sci. USA.

[212] Gerber P.A., Rutter G.A. (2017). The Role of Oxidative Stress and Hypoxia in Pancreatic Beta-Cell Dysfunction in Diabetes Mellitus. Antioxid. Redox Signal..

[213] Kaneto H., Fujii J., Seo H.G., Suzuki K., Matsuko T.-A., Masahiro N., Tatsumi H., Yamasaki Y., Kamada T., Taniguchi N. (1995). Apoptotic Cell Death Triggered by Nitric Oxide in Pancreatic—Cells. Diabetes.

[214] Hong K., Xu G., Grayson T.B., Shalev A. (2016). Cytokines Regulate β-Cell Thioredoxin-interacting Protein (TXNIP) via Distinct Mechanisms and Pathways. J. Biol. Chem..

[215] Spindel O.N., World C., Berk B.C. (2012). Thioredoxin Interacting Protein: Redox Dependent and Independent Regulatory Mechanisms. Antioxid. Redox Signal..

[216] Cha-Molstad H., Saxena G., Chen J., Shalev A. (2009). Glucose-stimulated Expression of Txnip Is Mediated by Carbohydrate Response Element-binding Protein, p300, and Histone H4 Acetylation in Pancreatic Beta Cells. J. Biol. Chem..

[217] Chen J., Hui S.T., Couto F.M., Mungrue I., Davis D.B., Attie A.D., Lusis A.J., Davis R.A., Shalev A. (2008). Thioredoxin-interacting protein deficiency induces Akt/Bcl-xL signaling and pancreatic beta-cell mass and protects against diabetes. FASEB J..

[218] Chen J., Fontes G., Saxena G., Poitout V., Shalev A. (2009). Lack of TXNIP Protects Against Mitochondria-Mediated Apoptosis but Not Against Fatty Acid–Induced ER Stress–Mediated β-Cell Death. Diabetes.

[219] Lenzen S., Drinkgern J., Tiedge M. (1996). Low antioxidant enzyme gene expression in pancreatic islets compared with various other mouse tissues. Free. Radic. Biol. Med..

[220] Pullen T., Rutter G.A. (2012). When less is more: The forbidden fruits of gene repression in the adult β-cell. Diabetes Obes. Metab..

[221] Malaisse W.J., Malaisse-Lagae F., Sener A., Pipeleers D.G. (1982). Determinants of the selective toxicity of alloxan to the pancreatic B cell. Proc. Natl. Acad. Sci. USA.

[222] Xu J., Long Y.-S., Gozal D., Epstein P.N. (2008). β-cell death and proliferation after intermittent hypoxia: Role of oxidative stress. Free. Radic. Biol. Med..

[223] Grankvist K., Marklund S., Täljedal I.-B. (1981). Superoxide dismutase is a prophylactic against alloxan diabetes. Nature.

[224] Grankvist K., Marklund S.L., Täljedal I.B. (1981). CuZn-superoxide dismutase, Mn-superoxide dismutase, catalase and glutathione peroxidase in pancreatic islets and other tissues in the mouse. Biochem. J..

[225] Johansson L.H., Borg L.A.H. (1988). A spectrophotometric method for determination of catalase activity in small tissue samples. Anal. Biochem..

[226] Tersey S.A., Maier B., Nishiki Y., Maganti A.V., Nadler J.L., Mirmira R.G. (2014). 12-Lipoxygenase Promotes Obesity-Induced Oxidative Stress in Pancreatic Islets. Mol. Cell. Biol..

[227] Hernandez-Perez M., Chopra G., Fine J., Conteh A.M., Anderson R.M., Linnemann A.K., Benjamin C., Nelson J.B., Benninger K.S., Nadler J.L. (2017). Inhibition of 12/15-Lipoxygenase Protects Against β-Cell Oxidative Stress and Glycemic Deterioration in Mouse Models of Type 1 Diabetes. Diabetes.

[228] Prentki M., Corkey B.E. (1996). Are the beta-Cell Signaling Molecules Malonyl-CoA and Cystolic Long-Chain Acyl-CoA Implicated in Multiple Tissue Defects of Obesity and NIDDM?. Diabetes.

[229] Robertson R.P., Zhang H.J., Pyzdrowski K.L., Walseth T.F. (1992). Preservation of insulin mRNA levels and insulin secretion in HIT cells by avoidance of chronic exposure to high glucose concentrations. J. Clin. Investig..

[230] Olson L.K., Redmon J.B., Towle H.C., Robertson R.P. (1993). Chronic exposure of HIT cells to high glucose concentrations paradoxically decreases insulin gene transcription and alters binding of insulin gene regulatory protein. J. Clin. Investig..

[231] Gleason C.E., Gonzalez M., Harmon J.S., Robertson R.P. (2000). Determinants of glucose toxicity and its reversibility in the pancreatic islet β-cell line, HIT-T. Am. J. Physiol. Endocrinol. Metab..

[232] Harmon J.S., Stein R., Robertson R.P. (2005). Oxidative Stress-mediated, Post-translational Loss of MafA Protein as a Contributing Mechanism to Loss of Insulin Gene Expression in Glucotoxic Beta Cells. J. Biol. Chem..

[233] Boland B.B., Brown C., Boland M.L., Cann J., Sulikowski M., Hansen G., Grønlund R.V., King W., Rondinone C., Trevaskis J. (2018). Pancreatic β-Cell Rest Replenishes Insulin Secretory Capacity and Attenuates Diabetes in an Extreme Model of Obese Type 2 Diabetes. Diabetes.

[234] Elks M.L. (1993). Chronic perifusion of rat islets with palmitate suppresses glucose-stimulated insulin release. Endocrinology.

[235] Zhou Y.P., Grill V.E. (1994). Long-term exposure of rat pancreatic islets to fatty acids inhibits glucose-induced insulin secretion and biosynthesis through a glucose fatty acid cycle. J. Clin. Investig..

[236] Carpentier A., Mittelman S.D., Lamarche B., Bergman R.N., Giacca A., Lewis G.F. (1999). Acute enhancement of insulin secretion by FFA in humans is lost with prolonged FFA elevation. Am. J. Physiol. Endocrinol. Metab..

[237] Lupi R., Dotta F., Marselli L., Del Guerra S., Masini M., Santangelo C., Patané G., Boggi U., Piro S., Anello M. (2002). Prolonged Exposure to Free Fatty Acids Has Cytostatic and Pro-Apoptotic Effects on Human Pancreatic Islets: Evidence that -Cell Death Is Caspase Mediated, Partially Dependent on Ceramide Pathway, and Bcl-2 Regulated. Diabetes.

[238] Shimabukuro M., Zhou Y.T., Levi M., Unger R.H. (1998). Fatty acid-induced beta cell apoptosis: A link between obesity and diabetes. Proc. Natl. Acad. Sci. USA.

[239] Cnop M., Hannaert J.C., Grupping A.Y., Pipeleers D.G. (2002). Low Density Lipoprotein Can Cause Death of Islet β-Cells by Its Cellular Uptake and Oxidative Modification. Endocrinology.

[240] El-Assaad W., Buteau J., Peyot M.-L., Nolan C., Roduit R., Hardy S., Joly E., Dbaibo G., Rosenberg L., Prentki M. (2003). Saturated Fatty Acids Synergize with Elevated Glucose to Cause Pancreatic β-Cell Death. Endocrinology.

[241] Kharroubi I., Ladrière L., Cardozo A.K., Dogusan Z., Cnop M., Eizirik D.L. (2004). Free Fatty Acids and Cytokines Induce Pancreatic β-Cell Apoptosis by Different Mechanisms: Role of Nuclear Factor-κB and Endoplasmic Reticulum Stress. Endocrinology.

[242] Choi S.-E., Kim H.-E., Shin H.-C., Jang H.-J., Lee K.-W., Kim Y., Kang S.S., Chun J., Kang Y. (2007). Involvement of Ca2+-mediated apoptotic signals in palmitate-induced MIN6N8a beta cell death. Mol. Cell. Endocrinol..

[243] Ameisen J.C. (2002). On the origin, evolution, and nature of programmed cell death: A timeline of four billion years. Cell Death Differ..

[244] Buja L.M., Eigenbrodt M.L., Eigenbrodt E.H. (1993). Apoptosis and necrosis. Basic types and mechanisms of cell death. Arch. Pathol. Lab. Med..

[245] Gerschenson L.E., Rotello R.J. (1992). Apoptosis: A different type of cell death. FASEB J..

[246] Hui H., Dotta F., Di Mario U., Perfetti R. (2004). Role of caspases in the regulation of apoptotic pancreatic islet beta-cells death. J. Cell. Physiol..

[247] Marchetti P., Del Guerra S., Marselli L., Lupi R., Masini M., Pollera M., Bugliani M., Boggi U., Vistoli F., Mosca F. (2004). Pancreatic Islets from Type 2 Diabetic Patients Have Functional Defects and Increased Apoptosis That Are Ameliorated by Metformin. J. Clin. Endocrinol. Metab..

[248] Nagata S. (2010). Apoptosis and autoimmune diseases. Ann. N. Y. Acad. Sci..

[249] Segawa K., Nagata S. (2015). An Apoptotic ‘Eat Me’ Signal: Phosphatidylserine Exposure. Trends Cell Biol..

[250] Tonnus W., Belavgeni A., Beuschlein F., Eisenhofer G., Fassnacht M., Kroiss M., Krone N.P., Reincke M., Bornstein S.R., Linkermann A. (2021). The role of regulated necrosis in endocrine diseases. Nat. Rev. Endocrinol..

[251] Rongvaux A., Jackson R., Harman C.C., Li T., West A.P., De Zoete M.R., Wu Y., Yordy B., Lakhani S., Kuan C.-Y. (2014). Apoptotic Caspases Prevent the Induction of Type I Interferons by Mitochondrial DNA. Cell.

[252] Contreras C.J., Lin L., Hogan M.F., Oberst A., Kahn S.E., Templin A.T. (2021). 294-OR: RIPK3-Mediated Necroptosis Is an Alternative Form of TNFa-Induced ß-Cell Death. Diabetes.

[253] Proskuryakov S.Y., Konoplyannikov A.G., Gabai V.L. (2003). Necrosis: A specific form of programmed cell death?. Exp. Cell Res..

[254] Rock K.L., Kono H. (2008). The Inflammatory Response to Cell Death. Annu. Rev. Pathol. Mech. Dis..

[255] Fehsel K., Kolb-Bachofen V., Kröncke K.-D. (2003). Necrosis is the predominant type of islet cell death during development of insulin-dependent diabetes mellitus in BB rats. Lab. Investig..

[256] Steer S.A., Scarim A.L., Chambers K.T., Corbett J.A. (2005). Interleukin-1 Stimulates β-Cell Necrosis and Release of the Immunological Adjuvant HMGB. PLoS Med..

[257] Ling Z., Van De Casteele M., Eizirik D.L., Pipeleers D.G. (2000). Interleukin-1beta-induced alteration in a beta-cell phenotype can reduce cellular sensitivity to conditions that cause necrosis but not to cytokine-induced apoptosis. Diabetes.

[258] Hoorens A., Stangé G., Pavlovic D., Pipeleers D. (2001). Distinction Between Interleukin-1-Induced Necrosis and Apoptosis of Islet Cells. Diabetes.

[259] Bruni A., Pepper A.R., Pawlick R.L., Gala-Lopez B., Gamble A.F., Kin T., Seeberger K., Korbutt G.S., Bornstein S.R., Linkermann A. (2018). Ferroptosis-inducing agents compromise in vitro human islet viability and function. Cell Death Dis..

[260] Zheng J., Conrad M. (2020). The Metabolic Underpinnings of Ferroptosis. Cell Metab..

[261] Templin A.T., Hogan M.F., Esser N., Zraika S., Hull R.L., Kahn S.E. (2018). Evidence for Necroptosis as a Mechanism of Islet Amyloid–Induced Beta-Cell Death. Diabetes.

[262] Sha W., Hu F., Xi Y., Chu Y., Bu S. (2021). Mechanism of Ferroptosis and Its Role in Type 2 Diabetes Mellitus. J. Diabetes Res..

[263] Jehn M., Clark J.M., Guallar E. (2004). Serum Ferritin and Risk of the Metabolic Syndrome in U.S. Adults. Diabetes Care.

[264] Vandenabeele P., Galluzzi L., Berghe T.V., Kroemer G. (2010). Molecular mechanisms of necroptosis: An ordered cellular explosion. Nat. Rev. Mol. Cell Biol..

[265] Linkermann A., Green D.R. (2014). Necroptosis. N. Engl. J. Med..

[266] Yang B., Maddison L.A., Zaborska K.E., Dai C., Yin L., Tang Z., Zang L., Jacobson D.A., Powers A.C., Chen W. (2020). RIPK3-mediated inflammation is a conserved β cell response to ER stress. Sci. Adv..

[267] Kaczmarek A., Vandenabeele P., Krysko D. (2013). Necroptosis: The Release of Damage-Associated Molecular Patterns and Its Physiological Relevance. Immunity.

[268] Steinke J., Taylor K.W. (1974). Viruses and the Etiology of Diabetes. Diabetes.

[269] Filippi C.M., von Herrath M.G. (2008). Viral Trigger for Type 1 Diabetes: Pros and Cons. Diabetes.

[270] Cain H., Gerstenkorn B. (1962). Karyological, karyometric and histochemical studies on cell death and necrosis in liver implant in rats. Beitr. Pathol. Anat..

[271] Patrlck R.I., Kroe D.J., Klavins J.V. (1964). Renal papillary necrosis induced by heterologous serum. Arch. Pathol..

[272] Kerr J.F.R., Wyllie A.H., Currie A.R. (1972). Apoptosis: A Basic Biological Phenomenon with Wide-ranging Implications in Tissue Kinetics. Br. J. Cancer.

[273] Majno G., Joris I. (1995). Apoptosis, oncosis, and necrosis. An overview of cell death. Am. J. Pathol..

[274] Alberts B., Johnson A., Lewis J., Raff M., Roberts K., Walter P. Programmed Cell Death (Apoptosis). *Mol. Biol. Cell 4th Ed*. https://www.ncbi.nlm.nih.gov/books/NBK26873/.

[275] Elmore S. (2007). Apoptosis: A review of programmed cell death. Toxicol. Pathol..

[276] Old L.J. (1985). Tumor Necrosis Factor (TNF). Science.

[277] Jorgensen M.B. (1993). The role of signal transduction in the delayed necrosis of the hippocampal CA1 pyramidal cells following transient ischemia. Acta Neurol. Scand. Suppl..

[278] Kitanaka C., Kuchino Y. (1999). Caspase-independent programmed cell death with necrotic morphology. Cell Death Differ..

[279] Kraupp B.G., Ruttkay-Nedecky B., Koudelka H., Bukowska K., Bursch W., Schulte-Hermann R. (1995). In situ detection of fragmented dna (tunel assay) fails to discriminate among apoptosis, necrosis, and autolytic cell death: A cautionary note. Hepatology.

[280] Kelly K.J., Sandoval R.M., Dunn K.W., Molitoris B.A., Dagher P.C. (2003). A novel method to determine specificity and sensitivity of the TUNEL reaction in the quantitation of apoptosis. Am. J. Physiol. Physiol..

[281] Atale N., Gupta S., Yadav U., Rani V. (2014). Cell-death assessment by fluorescent and nonfluorescent cytosolic and nuclear staining techniques—ATALE—2014. J. Microsc..

[282] Kroemer G., Galluzzi L., Vandenabeele P., Abrams J., Alnemri E.S., Baehrecke E.H., Blagosklonny M.V., El-Deiry W.S., Golstein P., Green D.R. (2009). Classification of cell death: Recommendations of the Nomenclature Committee on Cell Death. Cell Death Differ..

[283] Galluzzi L., Aaronson S.A., Abrams J., Alnemri E.S., Andrews D.W., Baehrecke E.H., Bazan N.G., Blagosklonny M.V., Blomgren K., Borner C. (2009). Guidelines for the use and interpretation of assays for monitoring cell death in higher eukaryotes. Cell Death Differ..

[284] Orozco S.L., Yatim N., Werner M.R., Tran H., Gunja S.Y., Tait S.W.G., Albert M.L., Green D.R., Oberst A. (2014). RIPK1 both positively and negatively regulates RIPK3 oligomerization and necroptosis. Cell Death Differ..

[285] Shi H., Kwok R.T.K., Liu J., Xing B., Tang B.Z., Liu B. (2012). Real-Time Monitoring of Cell Apoptosis and Drug Screening Using Fluorescent Light-Up Probe with Aggregation-Induced Emission Characteristics. J. Am. Chem. Soc..

[286] Zhang J., Wang X., Cui W., Wang W., Zhang H., Liu L., Zhang Z., Li Z., Ying G., Zhang N. (2013). Visualization of caspase-3-like activity in cells using a genetically encoded fluorescent biosensor activated by protein cleavage. Nat. Commun..

[287] Khanna D., Hamilton C.A., Bhojani M.S., Lee K.C., Dlugosz A., Ross B.D., Rehemtulla A. (2010). A Transgenic Mouse for Imaging Caspase-Dependent Apoptosis within the Skin. J. Investig. Dermatol..

[288] Tang H.L., Fung M.C., Hardwick J.M. (2015). In vivo CaspaseTracker biosensor system for detecting anastasis and non-apoptotic caspase activity. Sci. Rep..

[289] Murai S., Yamaguchi Y., Shirasaki Y., Yamagishi M., Shindo R., Hildebrand J.M., Miura R., Nakabayashi O., Totsuka M., Tomida T. (2018). A FRET biosensor for necroptosis uncovers two different modes of the release of DAMPs. Nat. Commun..

